# HTLV-1 bZIP Factor Impairs Anti-viral Immunity by Inducing Co-inhibitory Molecule, T Cell Immunoglobulin and ITIM Domain (TIGIT)

**DOI:** 10.1371/journal.ppat.1005372

**Published:** 2016-01-06

**Authors:** Keiko Yasuma, Jun-ichirou Yasunaga, Keiko Takemoto, Kenji Sugata, Yuichi Mitobe, Norihiro Takenouchi, Masanori Nakagawa, Yutaka Suzuki, Masao Matsuoka

**Affiliations:** 1 Laboratory of Virus Control, Institute for Virus Research, Kyoto University, Sakyo-ku, Kyoto, Japan; 2 Laboratory of Biological Protection, Institute for Virus Research, Kyoto University, Sakyo-ku, Kyoto, Japan; 3 Department of Microbiology, Kansai Medical University, Hirakata, Osaka, Japan; 4 North Medical Center, Kyoto Prefectural University of Medicine, Yosano-cho, Kyoto, Japan; 5 Department of Computational Biology and Medical Science, Graduate School of Frontier Sciences, The University of Tokyo, Kashiwa, Chiba, Japan; University of Pennsylvania School of Medicine, UNITED STATES

## Abstract

Human T-cell leukemia virus type 1 (HTLV-1) infects CD4^+^ T cells and induces proliferation of infected cells *in vivo*, which leads to the onset of adult T-cell leukemia (ATL) in some infected individuals. The HTLV-1 bZIP factor (*HBZ*) gene, which is encoded in the minus strand of HTLV-1, plays critical roles in pathogenesis. In this study, RNA-seq and ChIP-seq analyses using HBZ transduced T cells revealed that HBZ upregulates the expression and promoter acetylation levels of a co-inhibitory molecule, T cell immunoglobulin and ITIM domain (*TIGIT*), in addition to those of regulatory T cells related genes, *Foxp3* and *Ccr4*. TIGIT was expressed on CD4^+^ T cells from HBZ-transgenic (HBZ-Tg) mice, and on ATL cells and HTLV-1 infected CD4^+^ T cells of HTLV-1-associated myelopathy/tropical spastic paraparesis (HAM/TSP) *in vivo*. Expression of Blimp1 and IL-10 was upregulated in TIGIT^+^CD4^+^ cells of HBZ-Tg mice compared with TIGIT^-^CD4^+^ T cells, suggesting the correlation between TIGIT expression and IL-10 production. When CD4^+^ T cells from HBZ-Tg mice were stimulated with TIGIT’s ligand, CD155, their production of the inhibitory cytokine IL-10 was enhanced. Furthermore, dendritic cells from HBZ-Tg mice produced high levels of IL-10 after stimulation. These data suggest that HBZ alters immune system to suppressive state via TIGIT and IL-10. Importantly, TIGIT suppressed T-cell responses to another HTLV-1 virus protein, Tax, *in vitro*. Blocking of TIGIT and PD-1 slightly increased anti-Tax T-cell activity in some HAM/TSP patients. These results suggest that HBZ-induced TIGIT on HTLV-1 infected cells impairs T-cell responses to viral antigens. This study shows that HBZ-induced TIGIT plays a pivotal role in attenuating host immune responses and shaping a microenvironment favorable to HTLV-1.

## Author Summary

HTLV-1 is a T-cell-tropic, latently infectious virus that causes a T-cell malignancy, ATL, and inflammatory diseases. The mechanisms by which HTLV-1 evades the immune response and establishes chronic infection are not yet understood. Recent studies have demonstrated that TIGIT, a co-inhibitory molecule, is expressed on tumor infiltrating T cells and T cells during viral infection, which suppresses the anti-tumor and anti-viral immune responses. Furthermore, blockade of co-inhibitory molecules of TIGIT and programmed cell death-1 (PD-1) disrupts immune checkpoints and enhances anti-tumor activity. We found that TIGIT is upregulated by HBZ, and TIGIT impairs anti-virus immune responses through an immunosuppressive cytokine, IL-10. These findings show that HTLV-1 utilizes a co-inhibitory molecule on infected cells to evade the host immune responses. We also found that blocking of TIGIT and PD-1 on peripheral blood mononuclear cells in HTLV-1 infected patients enhances immune responses to virus. These findings suggest a mechanism by which HTLV-1 shapes a microenvironment favorable to its persistence using induced TIGIT. TIGIT is a potential therapeutic target for ATL and HTLV-1 infected patients.

## Introduction

Oncogenic viruses, including Epstein-Barr virus (EBV), Kaposi’s sarcoma-associated herpesvirus (KSHV), human papilloma virus (HPV), hepatitis B virus (HBV), hepatitis C virus (HCV), Merkel cell polyomavirus and human T-cell leukemia virus type 1 (HTLV-1), cause approximately 12% of human cancers. In these virus-induced cancers, a limited number of viral proteins play critical roles in oncogenesis—proteins that include HBx for HBV, E6 and E7 for HPV, and Tax and HTLV-1 bZIP factor (HBZ) for HTLV-1 [1]. These viral proteins influence a cell’s epigenetic status and/or modulate a cell’s transcriptional machinery, leading to the transformation of infected cells.

HTLV-1 causes adult T-cell leukemia (ATL) in a fraction of infected individuals after a long latent period [2]. HTLV-1 induces clonal proliferation of infected cells *in vivo* [3]. The *HBZ* gene, which is encoded in the minus strand, is expressed in all ATL cases and is reported to cause inflammation and T-cell lymphoma, and associate with latency [4–6]. However, the precise mechanism by which this occurs is not fully understood. HTLV-1 causes the proliferation of infected cells *in vivo*, but the host immune response influences the population dynamics of infected cells. One of the main issues for HTLV-1 pathogenesis is how HTLV-1 infected cells are enabled to evade the host immune response and establish the chronic infection. In HTLV-1 infected individuals, the provirus is mainly present in CD4^+^CCR4^+^CADM1^+^ T cells, indicating that this virus targets a certain subpopulation of T cells [7, 8]. Furthermore, this virus is frequently detected in Foxp3^+^ T cells *in vivo* [9]. Since HBZ enhances transcription of the *Foxp3* gene through enhanced TGF-β/Smad signaling [10], it is thought that HBZ alters the immunophenotype of infected cells. Although Foxp3 induction may affect the immune status of infected individuals, it is not yet certain how HTLV-1 causes immunosuppression in its hosts.

Members of the CD28 family, especially the co-stimulatory molecule CD28 and the co-inhibitory molecules CTLA-4 and PD-1, play important roles in regulating T-cell function [11, 12]. Several cancers have been shown to exploit such immune checkpoint pathways to evade the host immune responses; thus, blocking of these pathways is a promising new strategy for cancer therapy. Indeed, blocking antibodies have shown to be effective for melanoma and other cancers [13, 14]. Another inhibitory molecule of the CD28 family is T cell immunoglobulin and ITIM domain (TIGIT), which is expressed on activated T cells, regulatory T (Treg) cells, and NK cells. TIGIT binds to CD155 (also known as poliovirus receptor) and CD112 on dendritic cells (DCs), and TIGIT competes with a co-stimulatory receptor, CD226, for CD155 binding [15]. TIGIT suppresses T-cell proliferation and function by inhibiting the binding of CD155 to CD226, through an intrinsic inhibitory signal via the ITIM domain of TIGIT or by enhancing IL-10 production from DCs through a reverse signal via CD155 [16–18]. Furthermore, TIGIT on T cells inhibits T-cell responses that are implicated in anti-tumor and anti-viral immunity [19]. It has been clearly shown that TIGIT plays critical roles to control viral infection *in vivo* [19].

In this study, we analyzed epigenetic and transcriptional changes induced by HBZ, and we identified TIGIT as a target of HBZ. HBZ expression upregulated *IL-10* mRNA in TIGIT^+^CD4^+^ T cells. TIGIT was also implicated in enhanced IL-10 production from DCs. Furthermore, TIGIT was highly expressed on ATL cells and HTLV-1 infected cells from HTLV-1-associated myelopathy/tropical spastic paraparesis (HAM/TSP) patients. TIGIT-Fc could suppress T-cell responses to a viral antigen, Tax, suggesting that HBZ-induced TIGIT is implicated in evasion of the host defense.

## Results

### Epigenetic and transcriptional changes induced by HBZ

**Fig 1:**
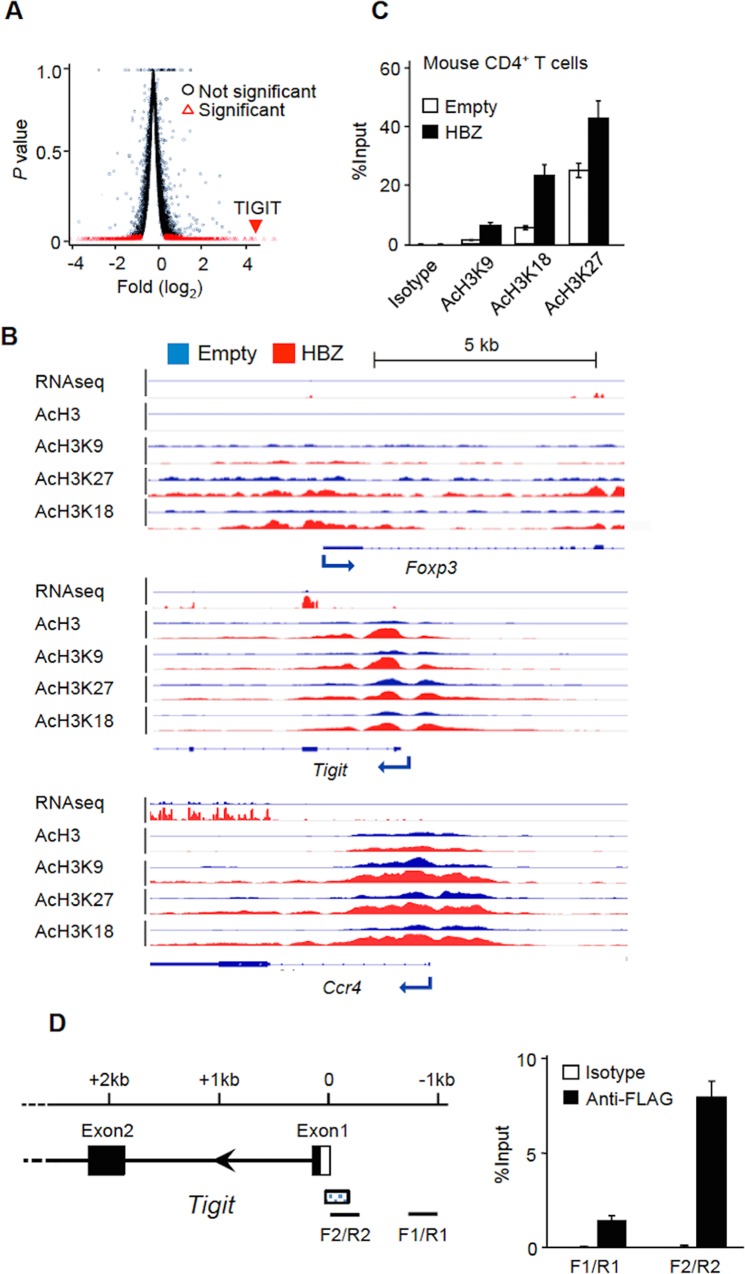
TIGIT transcription is regulated by HBZ. (A) Volcano plots of p-value (y-axis) against log_2_ fold change (x-axis: HBZ expressing murine primary T cells vs. control cells) for RNA-seq data. (B) RNA-seq and ChIP-seq results of three Treg related genes. RNA-seq was performed twice and ChIP-seq was performed once for each. (C) The enrichment of the *TIGIT* promoter region by the indicated histone marks or isotype control was measured by ChIP-qPCR. The % input of HBZ expressing murine primary CD4^+^ T cells or control cells is shown. (D) The enrichment of the TIGIT promoter regions by anti-FLAG was analyzed by ChIP-seq in HBZ-FLAG expressing cells. The detected peak in the TIGIT promoter region was shown as the dotted box. The enrichment of the TIGIT promoter regions by anti-FLAG or isotype control was measured by ChIP-qPCR using the primers indicated. Results shown are the mean ± SD in triplicate (C, D).

To analyze transcriptional and epigenetic changes induced by HBZ, we transduced retroviruses expressing HBZ and GFP into mouse primary CD4^+^ T cells. Our previous studies showed that HBZ expression in mouse T cells induced immunophenotypic changes, including Foxp3 expression and effector/memory T-cell phenotype, similar to those in human HTLV-1 infected cells [5]. After sorting GFP^+^ cells, we analyzed the transcriptome of HBZ expressing CD4^+^ T cells by RNA-seq. We found that 12,620 genes were expressed at > 1 reads per kilobase of exon per million mapped reads (RPKM) in two independent experiments. To identify HBZ-regulated genes, we selected differentially expressed genes, which showed a higher or lower expression in HBZ expressing samples compared to the control (Fig 1A). We narrowed down our focus to genes with ≥ 4 times or ≤ 1/16 times the expression level of the control. We thus identified 150 genes upregulated and 68 genes suppressed by HBZ (S1 and S2 Tables). Genes upregulated by HBZ with log_2_ fold change >2.9 are shown in S1 Table. Upregulated genes included *Foxp3*, *TIGIT*, *PD-1*, *IL-10*, *IL-4*, *CCR4*, and *NRP1* genes.

We also assessed the association of the transcriptome with epigenetic modifications induced by HBZ by performing Chromatin immunoprecipitation (ChIP)-seq for 4 histone modifications: H3ac (H3 pan-acetyl), H3K9ac, H3K27ac, and H3K18ac. We found about 11,000 binding peaks of ChIP-seq tags in each sample, and peaks around transcription start site (TSS) were analyzed (S3 Table). The peaks for the acetylation of pan-H3 and specific lysine residues of H3 were associated with genes as follows; 43.3% and 44.2% of peaks of H3ac in HBZ and control CD4^+^ T cells; 52.9% (HBZ) and 46.9% (control) peaks of H3K9ac, 32.8% (HBZ) and 35.6% (control) peaks of H3K27ac, and 28.2% (HBZ) and 28.1% (control) peaks of H3K18ac, respectively. Correlations between the expression of mRNA and the alteration of histone modifications were observed in several Treg cells associated genes including *Foxp3*, *TIGIT* and *Ccr4* (Fig 1B) [10]. All histone acetylation marks showed similar pattern, and were correlated to the transcription. Among these genes, *TIGIT* was prominently upregulated by HBZ. Furthermore, the acetylation levels of H3K9, H3K18 and H3K27 of the *TIGIT* promoter were increased as shown by conventional ChIP-quantitative polymerase chain reaction (ChIP-qPCR) (Fig 1C). Taken together, these results indicate that HBZ upregulates the *TIGIT* transcription.

To explore whether HBZ itself interacts with the *TIGIT* associated genome, we performed ChIP-seq analysis with ectopic 3xFLAG-HBZ fusion protein expressing mouse CD4^+^ T cells using FLAG antibody. ChIP-seq tag peaks were analyzed when they were located around TSS. 201 peaks were identified in HBZ-FLAG expressing CD4^+^ T cells, 37 (18.4%) of which were associated with genes (S3 and S4 Tables). TIGIT was one of the regions significantly enriched by anti-FLAG while CCR4 and Foxp3 were not enriched in this study. We further confirmed that the promoter of *TIGIT* was enriched by anti-FLAG using the ChIP-qPCR (Fig 1D). These results suggested that HBZ was recruited to the *TIGIT* promoter, thereby inducing its transcription.

### HBZ induces TIGIT expression

**Fig 2:**
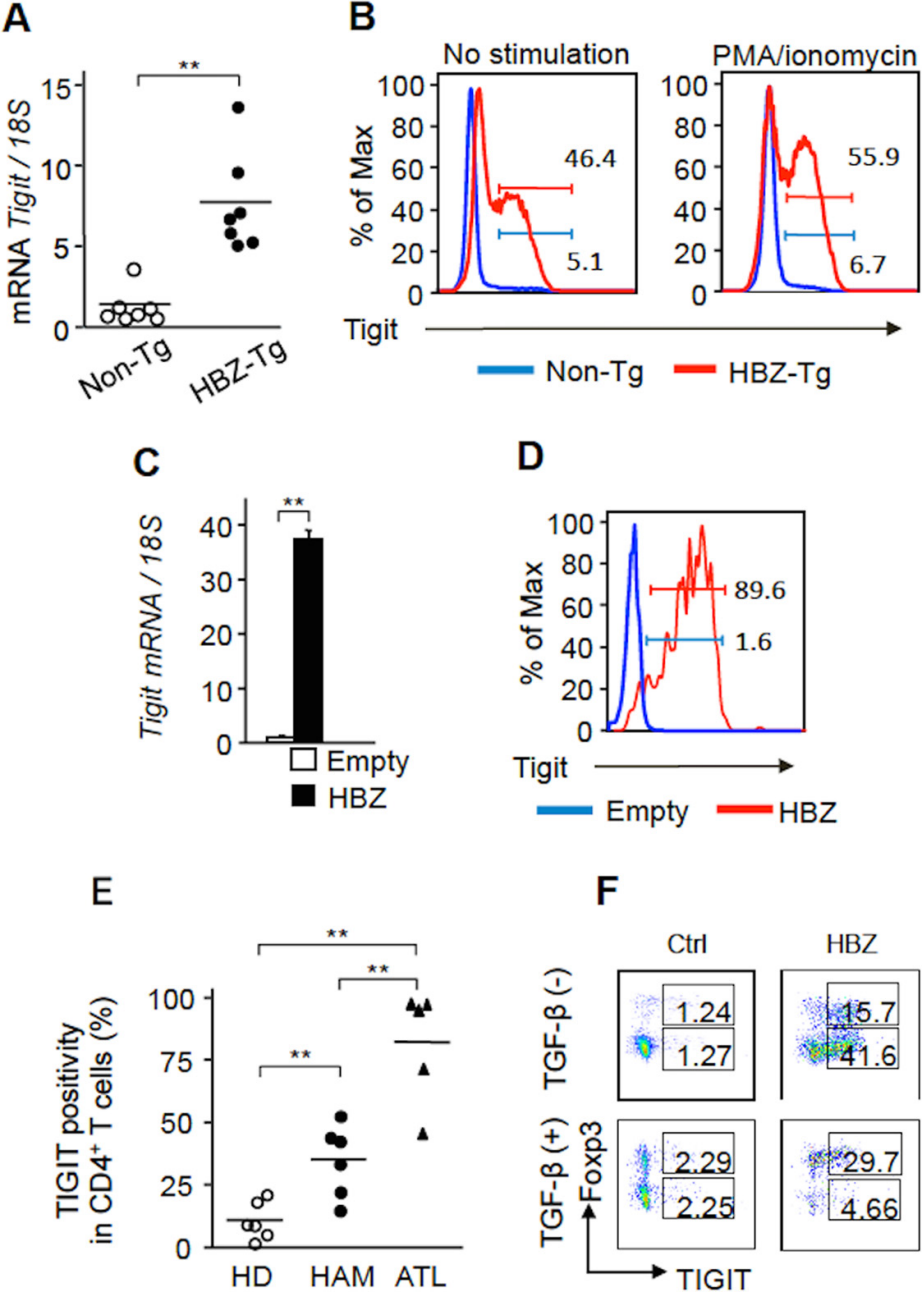
HBZ upregulates TIGIT expression. (A) *TIGIT* mRNA was increased in CD4^+^ T cells of HBZ-Tg mice (n = 7) compared with those of non-Tg mice (n = 7). (B) TIGIT expression was enhanced on resting and activated CD4^+^ T cells from HBZ-Tg mice by flow cytometry (FCM). The representative histogram of two independent experiments is shown. (C) A retrovirus expressing HBZ or control retrovirus was transduced into mouse primary CD4^+^ T cells. *TIGIT* transcripts were measured by realtime PCR. Results are the mean ± SD in triplicate. Three independent experiments were performed and the representative results are shown. (D) Expression of TIGIT on HBZ transduced mouse primary CD4^+^ T cells was measured by FCM. The representative result was shown for two independent experiments. (E) Expression of TIGIT on CD3^low^CD4^+^ T cells from ATL patients (n = 5) and CD4^+^CADM1^+^ T cells from HAM/TSP patients (n = 6) and healthy donors (HD, n = 6) were measured by FCM. (F) Expression of TIGIT on HBZ-transduced murine primary CD4^+^ cells and control cells in the presence or absence of TGF-β was measured by FCM. Two independent experiments were performed and the representative results are shown. *P < 0.05, **P < 0.01.

To study the effect of HBZ on TIGIT expression, we first analyzed TIGIT expression in HBZ-Tg mice. *TIGIT* mRNA was upregulated in CD4^+^ T cells from HBZ-Tg mice (Fig 2A). Furthermore, TIGIT expression was increased on resting and activated CD4^+^ T cells of HBZ-Tg mice by flow cytometry (Fig 2B). Expression of HBZ via a retrovirus vector in mouse CD4^+^ T cells also induced the *TIGIT* gene transcription (Fig 2C), and TIGIT expression on CD4^+^ T cells (Fig 2D).

Next we analyzed whether TIGIT is expressed on ATL cells and HTLV-1 infected cells. It has been reported that HTLV-1 infected cells are CADM1^+^ and ATL cells are CD3^low^CD4^+^ [8, 20, 21]. We analyzed TIGIT expression on these cells by flow cytometry. TIGIT expression was increased on both ATL cells and HTLV-1 infected cells of HAM/TSP patients (Fig 2E, Table 1). These findings suggest that HTLV-1 infection induces TIGIT expression. To check this possibility, human T cells were infected by HTLV-1 through co-culture with irradiated MT-2 cells. *De novo* infection also induced expression of TIGIT on infected cells (S1 Fig). Furthermore, to confirm whether HBZ is responsible for TIGIT expression, HBZ expression was suppressed by siRNA. Suppressed HBZ expression led to decreased TIGIT transcripts in ATL-43T cells (S2 Fig), suggesting that HBZ indeed induces TIGIT expression.

**Table 1:** Expression of TIGIT and PD-1 on ATL cells and HTLV-1 infected CD4+ T cells.*

Disease Type	TIGIT^-^ PD1^-^	TIGIT^+^ PD1^-^	TIGIT^-^ PD1^+^	TIGIT^+^ PD1^+^
ATL #1 (c)	0.06	0.61	1.91	97.4
ATL #2 (c)	7.38	4.38	19.6	68.6
ATL #3 (c)	0.31	21.3	0.15	78.3
ATL #4 (a)	51.2	39.8	2.66	6.38
ATL #5 (a)	2.69	1.76	1.76	93.8
HAM/TSP #1	17.8	18.9	12.0	51.3
HAM/TSP #2	34.5	30.2	14.3	21.1
HAM/TSP #3	13.2	19.9	15.6	51.2
HAM/TSP #4	18.1	13.6	12.6	55.7
HAM/TSP #5	20.8	24.1	11.9	43.2
Healthy donors	75.3 ± 8.91	3.09 ± 1.05	13.8 ± 3.73	7.89 ± 4.44

*For acute ATL cases, the numbers indicate the percentage of CD3^low^CD4^+^ ATL cells in each category. For HAM/TSP cases, the numbers indicate the percentage of CD4^+^CADM1^+^ T cells. In healthy donors, the numbers indicate the percentage of CD4^+^ T cells. The mean percentages (± SD) from 4 donors are shown for healthy donors.

(a): acute ATL,

(c): chronic ATL.

It has been reported that Foxp3 regulates TIGIT expression [22]. Since HBZ also induces transcription of the *Foxp3* gene through interaction with Smad2/3 and p300 [10], HBZ-induced Foxp3 might be implicated in TIGIT expression. When we transduced retrovirus expressing HBZ into mouse primary CD4^+^ T cells in the presence or absence of TGF-β, HBZ strongly induced Foxp3 expression in the presence of TGF-β. We found that HBZ induces TIGIT expression on both Foxp3^+^ and Foxp3^-^ CD4^+^ T cells (Fig 2F), indicating that HBZ can induce TIGIT expression regardless of Foxp3 expression.

**Fig 3:**
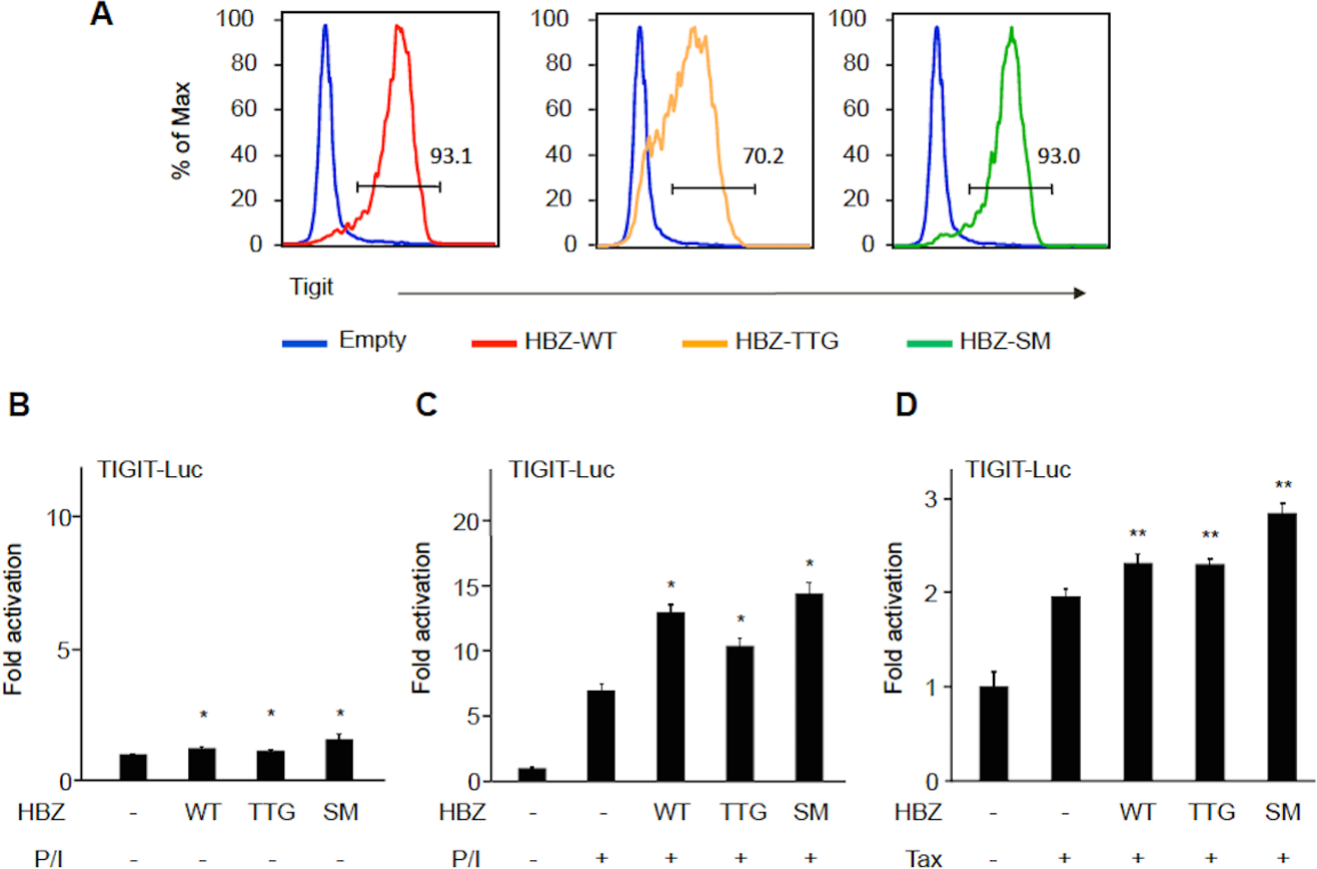
Effect of HBZ on the *TIGIT* gene promoter. (A) A retrovirus expressing wild-type HBZ, HBZ-RNA (TTG), HBZ-protein (SM) or control retrovirus was transduced into mouse primary CD4^+^ T cells. TIGIT expression on transduced T cells was analyzed by FCM. Two independent experiments were performed, and the representative results are shown. (B) *TIGIT*-promoter-Luc vector was transfected to Jurkat cells with wt or mutant HBZ. Luciferase activity was measured 24 hours after transfection. (C) *TIGIT*-promoter-Luc vector was transfected to Jurkat cells with 600 ng of wt or mutant HBZ. Luciferase activity was measured four hours after PMA/ionomycin (P/I) stimulation. (D) Jurkat cells were cotransfected with TIGIT-promoter-Luc, 300 ng of Tax expression vector, 100 ng of vectors expressing wt or mutant HBZ, and pGL4-TK. Two independent experiments were performed, and the representative results are shown. Results are the mean ± SD. *P < 0.05, **P < 0.01.

We have reported that both *HBZ* RNA and protein possess different effects on transcription of cellular genes [23, 24]. To study whether *HBZ* RNA and protein induces TIGIT expression on CD4^+^ T cells, recombinant retrovirus expressing wild-type (wt) or mutant HBZ and GFP was transduced into mouse activated CD4^+^ T cells. In the TTG mutant, the start codon ATG is replaced by TTG. Therefore, TTG mutant does not generate its protein. In the SM mutant, the entire coding region of HBZ is mutated with silent mutations, which indicates that the SM encodes the same protein while its RNA sequences and secondary structure are altered. Both TTG and SM mutant HBZ induced TIGIT expression on CD4^+^ T cells like wtHBZ (Fig 3A), indicating that both *HBZ* RNA and protein could induce TIGIT expression. Enhanced TIGIT expression was more potent by HBZ protein (SM) compared with *HBZ* RNA (TTG).

Next we examined the effect of HBZ on the promoter activity of *TIGIT*. wtHBZ and mutant HBZ slightly enhanced transcription from *TIGIT* promoter (Fig 3B). In the presence of phorbol myristate acetate (PMA)/ionomycin stimulation, both *HBZ* RNA and protein activated transcription from the *TIGIT* promoter (Fig 3C) although effect of HBZ protein was more potent than that of *HBZ* RNA. This finding is consistent with the result of Fig 3A. Furthermore, Tax activated transcription from *TIGIT* promoter, which was also augmented by HBZ (Fig 3D). Transcription level of HBZ expression vectors was analyzed by realtime PCR (S3 Fig), which showed that activation of *TIGIT* promoter is not caused by different transcription level of each expression vector. When recombinant retrovirus expressing HBZ was transduced, mouse T cells were activated by anti-CD3 antibody and antigen-presenting cells (APCs) since mouse retrovirus can infect only dividing cells. These findings suggest that HBZ activates the *TIGIT* gene transcription along with cell activation or Tax.

### HBZ enhances IL-10 production

**Fig 4:**
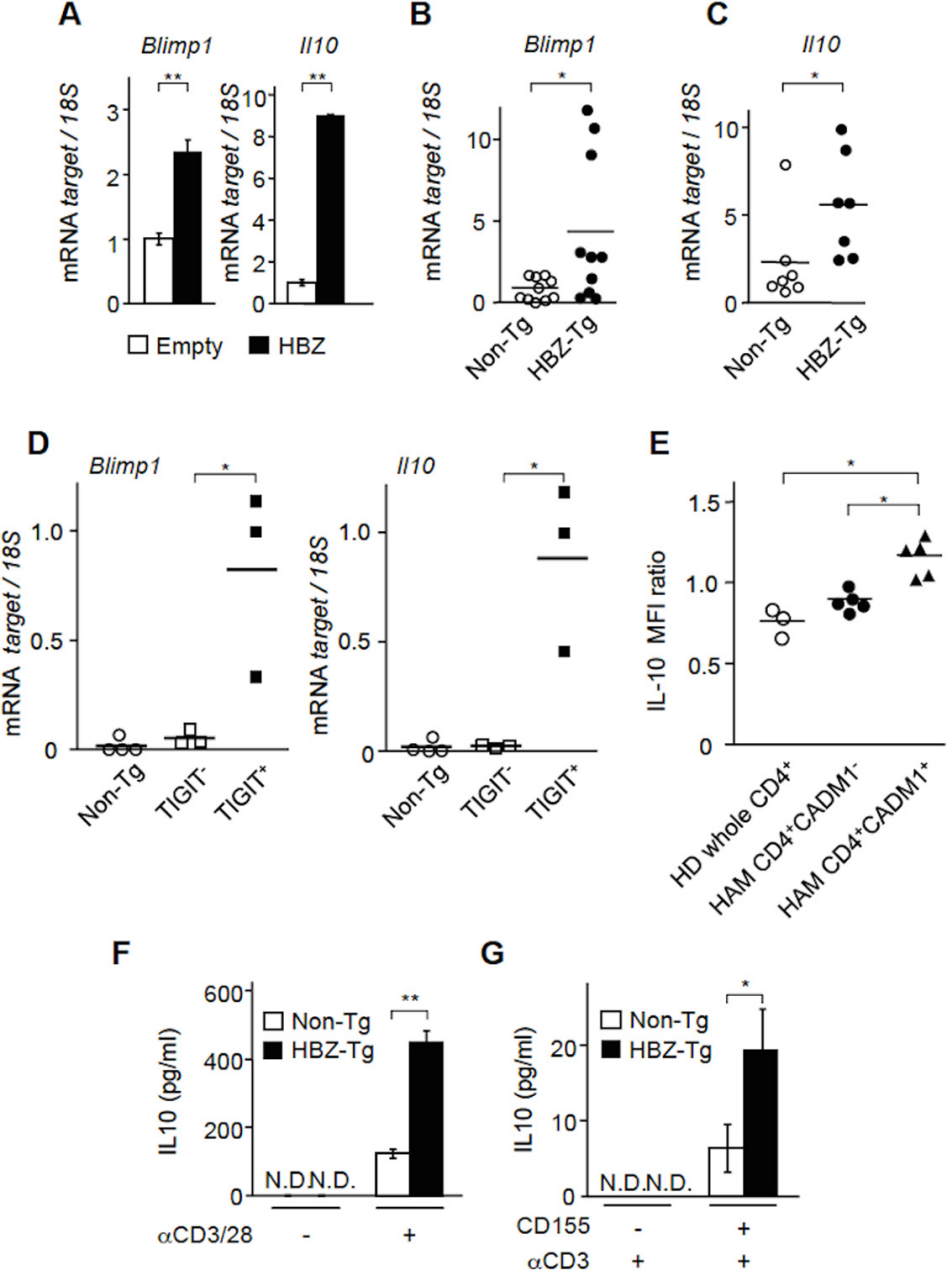
HBZ enhances IL-10 production. (A) The levels of *Blimp1* and *IL-10* mRNA were measured in HBZ-transduced murine primary CD4^+^ T cells and control cells by realtime PCR. This experiment was carried out in triplicate, and the mean values ± SD are shown. Three independent experiments were performed and the representative data are shown. (B) The levels of *Blimp1* were measured in CD4^+^ T cells from HBZ-Tg (n = 10) and non-Tg (n = 10) mice. (C) The levels of *IL-10* mRNA were measured in CD4^+^ T cells from HBZ-Tg (n = 7) and non-Tg (n = 7) mice using realtime PCR. (D) The levels of *Blimp1* and *IL-10* mRNA were measured in CD4^+^ T cells from non-Tg (n = 4) and TIGIT^+^CD4^+^ and TIGIT^-^CD4^+^ T cells from HBZ-Tg (n = 3) mice. (E) Expression of IL-10 was measured by FCM in CD4^+^ T cells from HD (n = 3) and CD4^+^CADM1^+^ and CD4^+^CADM1^-^ cells from HAM/TSP patients (n = 5). IL-10 MFI ratio to isotype control was analyzed. (F) CD4^+^ T cells from HBZ-Tg mice and from non-Tg mice were cultured with or without plate-coated anti-CD3 mAb (1 μg/ml) and soluble anti-CD28 mAb (1 μg/ml) for 24 hours. IL-10 production was measured by ELISA. N.D., not detected. (G) CD4^+^ T cells from HBZ-Tg mice and from non-Tg mice were cultured in the presence of plate-coated anti-CD3 mAb (1 μg/ml) with or without plate-coated CD155 (1 μg/ml) for 12 hours. Results shown are the mean ± SD. The representative result was shown from two independent experiments.*P < 0.05, **P < 0.01.

It has been reported that TIGIT^+^ Treg cells exhibit a more potent suppressive function than TIGIT^-^ Treg cells [25]. As the molecular basis of this stronger suppression, the immunosuppressive cytokine IL-10 and fibrinogen-like protein 2 (Fgl2) were identified. Expression of *Blimp1*, a transcription factor that transactivates *IL-10*, is upregulated in TIGIT^+^ Treg cells [26]. Therefore, we studied whether HBZ influences the expression of *Blimp1* and *Il-10*. When HBZ was expressed in mouse primary CD4^+^ T cells, both *Blimp1*and *Il-10* gene transcriptions were enhanced (Fig 4A). Furthermore, transcripts of these genes were upregulated in CD4^+^ T cells from HBZ-Tg mice compared to those of non-Tg mice (Fig 4B and 4C). Increased Blimp1^+^CD4^+^ T cells were confirmed in HBZ-Tg mice by flow cytometry (S4 Fig). To analyze correlation between expression of these genes and TIGIT, we sorted TIGIT^+^ and−subpopulation in CD4^+^ T cells of HBZ-Tg mice and analyzed transcription levels of *Blinp1* and *IL-10* genes. Level of the *Blinp1* and *IL-10* genes transcription of TIGIT^+^ T cells was much higher than those of TIGIT^-^ T cells and CD4^+^ T cells of non-Tg mice (Fig 4D), indicating the linkage between TIGIT expression and these genes. Next, we analyzed IL-10 production in HTLV-1 infected cells of HAM/TSP patients. Intracytoplasmic IL-10 was increased in CD4^+^CADM1^+^ T cells of HAM/TSP patients compared with CD4^+^CADM1^-^ T cells of HAM/TSP patients and CD4^+^ T cells of healthy donors after activation (Fig 4E, S5 Fig), suggesting that IL-10 production was enhanced in HTLV-1 infected T cells.

As shown in Fig 4A and 4D, HBZ enhanced *IL-10* transcription. Next we measured IL-10 protein level by T-cell activation. CD4^+^ T cells from HBZ-Tg secreted higher level of IL-10 by stimulation of anti-CD3 and anti-CD28 antibodies compared with those of non-Tg mice (Fig 4F). It has been reported that depletion of TIGIT suppresses IL-10 production, suggesting that TIGIT expression is closely linked with IL-10 [27]. Furthermore, this study also showed that only TIGIT^+^CD4^+^ T cells expressed higher level of IL-10 compared with TIGIT^-^CD4^+^ T cells. Therefore, we analyzed whether engagement of TIGIT by its ligand CD155 enhanced IL-10 production. IL-10 production by anti-CD3 antibody was analyzed in the presence or absence of CD155 [27]. As shown in Fig 4G, CD155 augmented IL-10 production in CD4^+^ T cells of HBZ-Tg mice although its secretion was not detected without CD155 stimulation at the protein level. Thus, HBZ expression itself induces IL-10 transcription likely through enhanced *Blimp1* transcription, and furthermore TIGIT-mediated signal also augments it. Since IL-10 is an immunosuppressive cytokine that enables cancer cells evade the host immune system [28], these effects of HBZ are implicated in evasion of host defense to infected cells.

### HBZ suppresses Fgl2 and CD226 expression

**Fig 5:**
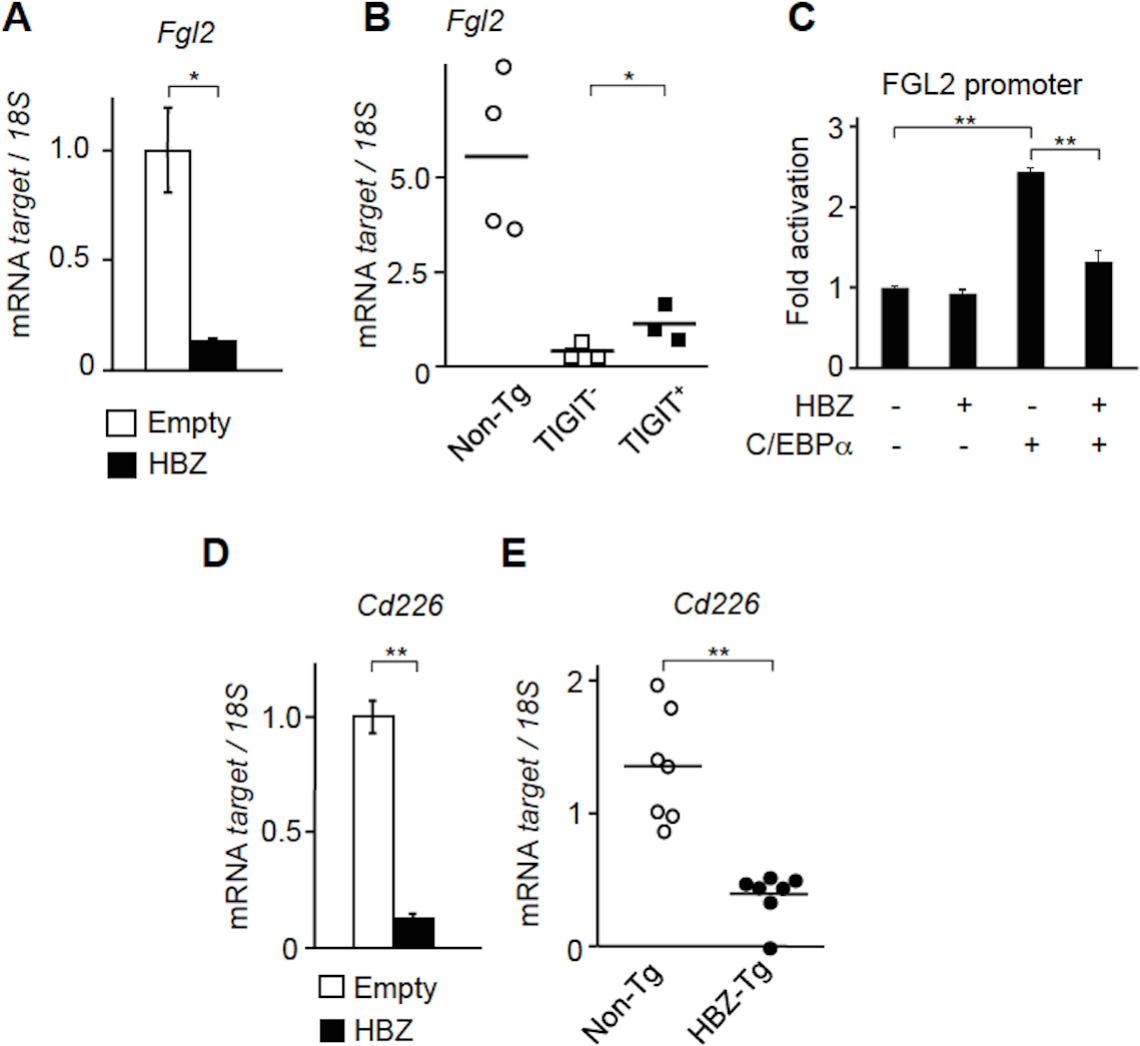
HBZ suppresses Fgl2 and CD226 expression. (A) *Fgl2* mRNA was quantified in HBZ-expressing murine primary CD4^+^ T cells and control cells by realtime PCR. This experiment was carried out in triplicate, and the mean values ± SD are shown. Three independent experiments were performed and the representative results are shown. (B) The levels of *Fgl2* mRNA were measured in CD4^+^ T cells from non-Tg (n = 4) and TIGIT^+^CD4^+^ and TIGIT^-^CD4^+^ T cells from HBZ-Tg (n = 3) mice. (C) Jurkat cells were cotransfected with 500 ng of FGL2-promoter-Luc, pME18Sneo-sHBZ, 500 ng of pcDNA-C/EBPα and pGL4-TK. Luciferase activity was measured after 24 hours. Luciferase assay was carried out in triplicate, and the mean values ± SD are shown. Three independent experiments were performed and the representative results were shown. (D) The *CD226* gene transcript was quantified in HBZ-expressing murine primary CD4^+^ T cells and control cells. This assay was carried out in triplicate and the mean values ± SD are shown. Three independent experiments were performed and the representative results were shown. (E) The *CD226* gene expression was measured in CD4^+^ T cells from HBZ-Tg (n = 7) and non-Tg (n = 7) mice by realtime PCR. *P < 0.05, **P < 0.01.

Fgl2 is a potent suppressive effector molecule of TIGIT^+^ Treg cells [25]. In contrast to *Il-10* and *Blimp1*, transcription of *Fgl2* was distinctly suppressed in HBZ expressing CD4^+^ T cells (Fig 5A). The *Fgl2* gene transcription was suppressed in both TIGIT^+^ and TIGIT^-^ T cells (Fig 5B), indicating that HBZ decreases this transcript regardless of TIGIT expression. Fgl2 transcript was not downregulated in ATL samples (S6 Fig).

C/EBPα is reported to promote transcription of *Fgl2* through binding to its genomic region [25]. We found that C/EBPα indeed activated the promoter of *Fgl2* and HBZ inhibited this C/EBPα-induced activation (Fig 5C). We measured transcription level of C/EBPα using realtime PCR, and found that HBZ did not influence transcription of C/EBPα (S7 Fig). This result is consistent with our previous report that HBZ suppresses C/EBPα signaling activation and the expression of its target genes by physical interference [29]. Thus, HBZ impairs the suppressive function of TIGIT^+^ Treg cells by interaction with C/EBPα leading to suppressed *Fgl2* transcription. This finding coincides with our previous report that HBZ induced Treg cells were functionally impaired by interaction between HBZ and Foxp3 [5], indicating that HBZ selectively modulates expression of immunosuppressive molecules in T cells.

TIGIT competes with a co-stimulatory receptor, CD226, for binding to CD155 and CD112 [16]. It has been reported that T-cell activation induces expression of both CD226 and TIGIT on CD4^+^ T cells [27]. Therefore, we next evaluated the effect of HBZ on *CD226* transcription. *CD226* mRNA was suppressed in HBZ expressing vector-transduced CD4^+^ T cells and CD4^+^ T cells from HBZ-Tg mice (Fig 5D and 5E), suggesting that HBZ inhibits transcription of the *CD226* gene and likely augments the function of TIGIT through suppression of its competitor. However, *CD226* transcripts were not always suppressed in ATL cases (S8 Fig), indicating that Tax expression and cell activation state of ATL cells might influence its transcription.

### HBZ modulates a DC phenotype by TIGIT on CD4^+^ T cells

**Fig 6:**
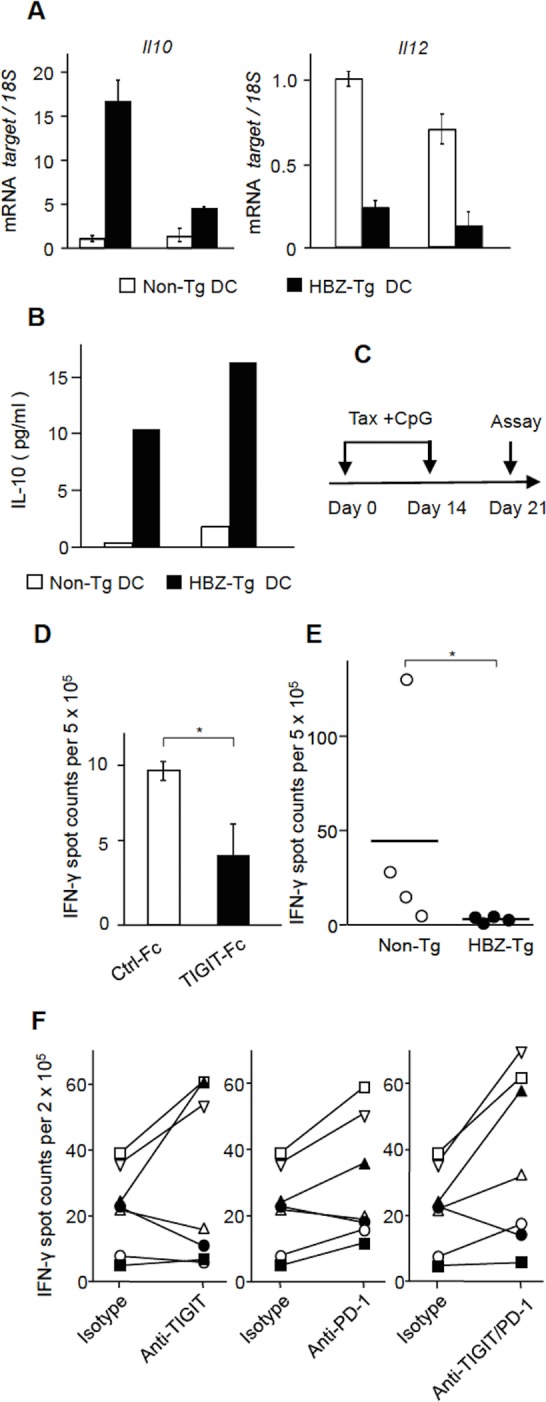
TIGIT suppresses T-cell response to Tax. (A) The production of IL-10 and IL-12 by DCs was modulated in HBZ-Tg mice. Splenocytes from HBZ-Tg mice and non-Tg mice were stimulated by LPS for 12 hours, and then DCs were sorted by FACS Aria II. The levels of *IL-10* and *IL-12* mRNA in DCs were measured by realtime PCR. Transcripts were quantified in triplicate and the mean values ± SD are shown. (B) Splenocytes from HBZ-Tg and non-Tg mice were stimulated by LPS overnight followed by sorting for DCs. Sorted cells were then cultured in the presence of LPS for 24 hours. The protein levels of IL-10 in cultured supernatants were measured by Cytometric Bead Array (CBA). Three independent experiments were performed and the representative result was shown. (C) Mice were immunized with Tax protein and CpG at day 0 and 14, and then sacrificed at day 21 for ELISPOT analysis. (D) Splenocytes from a Tax-immunized mouse were subjected to ELISPOT assay using Tax peptide stimulation along with TIGIT-Fc or control Fc conjugated beads. This assay was performed in triplicate and the results shown are the mean ± SD. The representative result was shown from two independent experiments. (E) HBZ-Tg mice (n = 4) and non-Tg mice (n = 4) were immunized by Tax protein and CpG, and then T-cell responses were measured by ELISPOT assay. (F) PBMCs from seven HAM/TSP patients (n = 7) were subjected to ELISPOT assay with Tax peptide stimulation in the presence or absence of anti-TIGIT and/or anti-PD-1 antibodies. The number of IFN-γ secreting cells normalized to the negative control is shown. Each symbol represented each patient. Results shown are the mean ± SD. *P < 0.05, **P < 0.01.

Binding of TIGIT to CD155 on DCs modulates them to acquire the immunomodulatory phenotype of increased IL-10 production and decreased IL-12p40 production–a DC phenotype that in turn inhibits T-cell activation [16]. Does this phenomenon occur in HBZ-expressing mice? We stimulated splenocytes from HBZ-Tg and non-Tg mice with LPS and then isolated DCs as CD11c^+^ cells. In HBZ-Tg mice, DCs do not express HBZ. *IL-10* transcript was remarkably increased and IL-12p40 production was severely suppressed in DCs from two HBZ-Tg mice (Fig 6A). Similarly, increased production of IL-10 from activated DCs was also confirmed by cytometric bead array (Fig 6B). These features are similar to those of DCs stimulated by TIGIT-Fc [16], suggesting that TIGIT on CD4^+^ T cells is implicated in changing the phenotype of DCs in these mice.

### Effects of TIGIT on anti-Tax T-cell responses

TIGIT on T cells is implicated in chronic viral infection [19]. This study also suggest that the induction of TIGIT on HBZ expressing CD4^+^ T cells modulates immune responses through increased production of IL-10 from both T cells and DCs—an effect that would likely generate a microenvironment advantageous to the persistence of HTLV-1 infected cells and ATL cells. To explore how TIGIT influences immune responses, we analyzed the effect of TIGIT-Fc on interferon-γ (IFN-γ) production of T cells stimulated by Tax peptides using enzyme-linked immunosorbent spot (ELISPOT) assay. C57BL/6J mice were immunized twice with recombinant Tax protein and CpG adjuvant (Fig 6C). Splenocytes were then stimulated with pooled Tax peptides along with beads conjugated with TIGIT-Fc or control-Fc. As shown in Fig 6D, the spots were decreased in the presence of TIGIT-Fc, indicating that TIGIT impairs anti-Tax T-cell response. When we immunized HBZ-Tg (n = 4) and non-Tg mice (n = 4) by Tax protein and CpG, the spots were severely suppressed in HBZ-Tg mice (Fig 6E), which indicates that T-cell responses to Tax are impaired in HBZ-Tg mice. Since HTLV-1 infected cells and ATL cells express high levels of TIGIT on their surfaces (Fig 2E), this data suggests that TIGIT on such cells may impair anti-Tax T-cell activity *in vivo*.

PD-1 is another co-inhibitory receptor expressed on T cells and a major target for immune checkpoint therapy. This study showed that *PD-1* transcript was upregulated by HBZ according to RNA-seq data (S1 Table), and PD-1 was expressed on HBZ expressing CD4^+^ T cells (S9 Fig), indicating that PD-1 is also a target of HBZ. We found that both TIGIT and PD-1 were expressed on most ATL cells and HTLV-1 infected cells of HAM/TSP patients (Table 1), as well as in CD8^+^ T cells of HAM/TSP patients (S5 Table). Since the positivity of TIGIT on CD8^+^ T cells is higher than proviral load, TIGIT expression on CD8^+^ T cells is likely induced by other mechanism than HTLV-1 infection. In consistent with this finding, increased PD-1 expression on CD8^+^ T cells of ATL patients was also reported [30]. Recently, monoclonal antibodies to PD-1 or its ligand, PD-L1, have exhibited clinical efficacy for patients with various cancers [15]. Furthermore, combined treatment with anti-PD-1 and anti-TIGIT antibodies significantly augments CTL activity against cancer cells [31]. Therefore, we analyzed the effects of anti-TIGIT and anti-PD-1 antibodies on the *in vitro* anti-Tax activity of T cells from HAM/TSP patients. As shown in Fig 6F, the spots of IFN-γ were increased by anti-TIGIT antibody in three cases, and by anti-PD-1 antibody in five cases and both antibodies in five cases. Thus, anti-TIGIT and PD-1 antibodies augmented anti-Tax T-cell responses in some HAM/TSP patients.

## Discussion

Antigen recognition by the T-cell receptor and a second signal mediated by the CD28-CD80/86 co-stimulatory pathway are critical for T-cell activation. In addition, a multitude of other co-stimulatory and co-inhibitory pathways are also involved in T-cell activation [12]. In particular, co-inhibitory receptors are pivotal in suppressing excess immune responses. Recently, antibodies to two co-inhibitory receptors, CTLA-4 and PD-1, called immune checkpoint blocking antibodies, have attracted attention as novel therapeutic drugs, since they exhibit remarkable clinical efficacy for patients with various cancers by reinvigorating exhausted CD8^+^ T cells [15]. Here we demonstrate that the HBZ induces TIGIT expression, which leads to increased production of IL-10 via T cells and DCs. Increased IL-10 production is likely associated with immunosuppressive effects on the host immune system. Furthermore, this study suggests that TIGIT suppresses anti-Tax T-cell responses *in vitro*. Antibodies to TIGIT and/or PD-1 enhanced anti-Tax T-cell response in peripheral blood mononuclear cells (PBMCs) of HAM/TSP patients, which suggests that TIGIT and PD-1 are targets of treatment for HTLV-1 associated diseases.

It has been reported that TIGIT plays critical roles to control viral infection [19]. In mice infected with lymphocytic choriomeningitis virus (LCMV), both TIGIT and PD-1 expressions were upregulated on CD4^+^ and CD8^+^ T cells. Viral loads were reduced and cytokine productions were increased in LCMV infected mice in which CD4^+^ T cells lack TIGIT expression. Furthermore, blockade of PD-1/PD-L1 and TIGIT by the antibodies synergistically enhanced viral clearance and CD8^+^ T cell effector functions. These data clearly indicates that TIGIT on T cells is implicated in anti-viral immunity. PD-1 and TIGIT of tumor infiltrating CD8^+^ T cells or exhausted CD8^+^ T cells in chronic infection have been extensively studied [19, 31]. Recently, it has been reported that melanoma cell intrinsic PD-1 promotes tumor growth [32]. In consistent with this report, this study also suggests that HBZ-induced TIGIT on ATL cells and infected cells is involved in pathogenesis of HTLV-1 infection. Thus, TIGIT on both infected cells and CD8^+^ effector T cells is implicated in evasion of the host defense.

As shown in this study, ATL cells and HTLV-1 infected cells express not only TIGIT but also PD-1 on their surfaces. HBZ can induce expression of both molecules. It has been reported that reverse signaling via PD-L1 and PD-L2 into DCs reduces DC maturation [33]. Furthermore, the binding of TIGIT to CD155 modulated cytokine production from monocyte-derived DCs by reverse signaling [16]. Similarly, the reverse signaling is a possible mechanism to increase IL-10 production and suppress IL-12p40 in stimulated DCs derived from HBZ-Tg mice. Recently, it has been reported that a combination of anti-PD-1 and anti-TIGIT antibodies remarkably restored the function of tumor antigen-specific CD8^+^ T cells, illustrating the significance of PD-1 and TIGIT on CD8^+^ T cells and the interactions of these receptors with their ligands on APCs and cancer cells [31]. The presence of anti-PD-1 and/or anti-TIGIT antibodies enhanced anti-Tax T-cell activity in this study, suggesting that these antibodies are clinically efficacious for the treatment of ATL patients.

Host immune responses play pivotal roles in controlling viruses. Accordingly, viruses acquire ways of counteracting host immune responses. For example, HCV NS3/4A cleaves IFN-β promoter stimulator-1, which blocks signaling via RIG-I/MDA5 [34]. Thus, HCV suppresses interferon production to escape innate immunity. Another example is that herpes viruses disturb antigen presentation by blocking the function of transporter associated with antigen processing (TAP) [35]. Likewise, HTLV-1 also has strategies for counteracting host immune surveillance. HTLV-1 p12 binds to human major histocompatibility complex class I (MHC-I) heavy chains, thereby decreasing MHC-I levels on infected cells [36]. This study suggests that TIGIT on HTLV-1 infected cells modulate the microenvironment, through increased production of IL-10, to suppress immune responses against viral antigens. Thus, HTLV-1 has evolved an elaborate strategy to evade the host immune system.

IL-10, a pleiotropic cytokine, exerts immunosuppressive functions on cytokine and chemokine production, suppresses MHC expression, and suppresses the maturation and function of DCs. It has been reported that KSHV microRNAs target C/EBPβ p20, thus inducing basal secretion of IL-6 and IL-10 by macrophages [37]. Viral homologues of IL-10 are encoded by EBV and CMV [38]. IL-10 and viral IL-10 diminish expression of MHC class II molecules on the monocytes, leading to impaired antigen-presenting capacity [39]. In HIV-1 infection, inflammatory cytokines enhance PD-1 expression on monocytes. Triggering of PD-1 by PD-L1 induces IL-10 production of monocytes, which dampen CD4^+^ T cell activation [40]. Thus, IL-10 is a key cytokine for controlling immune responses, and several viruses take advantage of this fact. The TIGIT-mediated upregulation of IL-10 production reported here is a novel mechanism for the survival of virus-infected cells.

HAM/TSP is an inflammatory disease of the central nervous system [41]. The immunosuppressive activity of HTLV-1-infected T cells as shown in this study might not coincide with this finding. Viral proteins of HTLV-1, HBZ and Tax, intrinsically induce inflammation. HBZ induces labile Foxp3 expression. Foxp3^+^CD4^+^ T cells that convert to Foxp3^-^CD4^+^ T cells produce excess amount of interferon-γ (IFN-γ), which leads to inflammation [42]. On the other hand, it has been reported that Tax increases T box transcription factor (T-bet), which promotes IFN-γ production [43]. Thus, both viral proteins induce inflammation. Although CTLs against Tax are also implicated in the pathogenesis of HAM/TSP, CTLs exclude HTLV-1 infected cells. TIGIT on HTLV-1 infected cells likely suppresses CTLs as shown in this study. Thus, TIGIT protects infected cells, which is supposed to promote onset of inflammatory diseases.

This study also illustrates epigenetic modulation by HBZ using genome-wide analysis. HBZ has been shown to interact with KIX domain of p300 [44]. We previously showed that HBZ interacts with host lysine acetylase, p300, and induces the expression of Foxp3 [10]. The adenovirus gene E1A is also reported to interact with p300/CBP and modulate the expression of p300-target host genes that inhibit viral replication [45]. Thus, adenovirus uses E1A to modify host histone-modifying enzymes to benefit its replication. In the present study, HBZ is shown to modulate the epigenetic state and induce the expression of several Treg related or inhibitory signal related genes, including *Foxp3*, *TIGIT*, and *CCR4* genes [25, 46]. It is assumed that HBZ generates a tumor- and virus- favorable microenvironment by modulating the transcription of cellular genes. The precise mechanism by which HBZ modulates epigenetic status of cellular genes remains to be studied.

In this study, we demonstrate that HBZ induced TIGIT expression likely impairs T-cell response to viral antigens through enhanced IL-10 production by T cells and DCs. This study suggests a new therapeutic strategy for ATL patients: the co-blockade of TIGIT and PD-1 to restore anti-tumor and anti-virus immune responses.

## Materials and Methods

### Animals and subjects

C57BL/6J mice were purchased from CLEA Japan. Transgenic mice expressing the *HBZ* gene in CD4^+^ T cells (HBZ-Tg) were described previously [23]. All mice (6–14 weeks of age) used in this study were maintained in an SPF facility. PBMCs of the patients with ATL or HAM/TSP and healthy donors were collected by Ficoll-Paque PLUS (GE Healthcare). Jurkat cell is a HTLV-1 negative human T-cell line, and MT-2 and 43T(-) cells are HTLV-1 positive human T-cell lines.

### Ethics statement

Animal experiments were performed in strict accordance with the Japanese animal welfare bodies (Law No. 105 dated 19 October 1973 modified on 2 June 2006), and the Regulation on Animal Experimentation at Kyoto University. The protocol was approved by the Institutional Animal Research Committee of Kyoto University (Permit numbers are D13-02, D14-02, and D15-02). Experiments using clinical samples were conducted according to the principles expressed in the Declaration of Helsinki, and approved by the Institutional Review Board of Kyoto University (Permit numbers are G310 and G204). ATL and HAM/TSP patients provided written informed consent for the collection of samples and subsequent analysis.

### Plasmids

Expression vectors for HBZ (wild-type and mutants) and C/EBPα were described previously [29, 47]. FLAG-HBZ was generated by inserting dimerized 3xFLAG oligos into the C-terminus of HBZ expressing retrovirus vector [5]. The promoter regions of *TIGIT* and *FGL2* were amplified from human genomic DNA by PCR using the primer sets described in S6 Table and cloned into pGL4.22 (Promega). The wild-type HBZ, HBZ RNA (TTG) and HBZ protein (SM) expressing vectors, and pCG-Tax were described [23].

### Retroviral transduction and RNA extraction

CD4^+^ cells were enriched by a CD4 enrichment kit (BD Pharmingen) and were activated by 0.5 μg/ml anti-CD3 Ab and 50 U/ml rIL-2 in the presence of T-cell-depleted and x-irradiated (20Gy) C57BL/6J splenocytes as APCs in 12 well plates. After 16 hours, activated T cells were transduced with viral supernatant and 4 μg/ml polybrene, and centrifuged at 3,000 rpm for 60 min. Cells were cultured in medium supplemented with 50 U/ml rIL-2 [5]. Two days after transduction, GFP expressing cells were sorted by FACS AriaII (BD). RNA was extracted using Trizol Reagent (Invitrogen).

### ChIP

Cells were transduced and sorted as described above, then crosslinked in 1% (0.5% for Fig 1C) formaldehyde solution and incubated for 10 min at room temperature. The remaining procedures were performed essentially as described [48] or using the SimpleChIP Enzymatic Chromatin IP Kit (Cell Signaling Technology) according to the manufacturer’s protocol.

### RNA-seq and ChIP-seq

We prepared RNA-seq libraries from the RNA described above and ChIP-seq libraries from chromatin-immunoprecipitated or input DNA samples, and we performed sequencing using the HiSeq2500 (Illumina) according to the manufacturer’s protocol. For HBZ-FLAG ChIP-seq analysis, library preparation and high-throughput sequencing were performed at BGI (Shenzhen, China) using the Hiseq2000 (Illumina).

### Mapping and analysis of RNA-seq and ChIP-seq

The obtained RNA-seq and ChIP-seq data were mapped to the murine reference genome using Bowtie [49]. For RNA-seq, differently expressing genes were analyzed using Cuffdiff and validated by realtime PCR using the primer sets described in S6 Table. Peaks of ChIP-seq tags were called by MACS1.4. Peaks located -2 kb to +0.5 kb of TSS were analyzed as peaks around TSS. RNA-seq and ChIP-seq files were visualized using the Integrative Genomics Viewer [50–52].

### Luciferase assay

Jurkat cells were seeded at 2 x 10^5^ cells/ml and transfected with 300 ng of luciferase reporter plasmid, 10 ng of pGL4-TK (Promega), and indicated amount of HBZ-expressing plasmid with or without Tax-expressing or C/EBPα-expressing plasmid using LTX (Invitrogen) according to the manufacturer’s protocol. After 24 hours, cells were harvested and luciferase activities were measured using the Dual Luciferase Reporter Assay Kit (Promega). Relative luciferase activities of Firefly to Renilla were then calculated. For PMA /ionomycin stimulation, cells were stimulated for 4 hours before being harvested. To compare the expression levels of HBZ mutants, transcripts in SRα region were quantified by realtime PCR.

### Flow cytometry

Human PBMCs and murine splenocytes were stained with the antibodies indicated according to the manufacturer’s protocol and analyzed using FACS Verse with Suite software (BD). Data was analyzed by FlowJo software (Treestar).

### Antibodies

ChIP-seq for histone modifications was performed using the following antibodies: anti-H3ac (06–599, Millipore) [53], anti-H3K9ac (07–352, Millipore) [54], and anti-H3K27ac (ab4729, Abcam) antibodies [48]. Anti-H3K18ac (39755, Active Motif) does not react to acetyl-Lys4, unmodified Lys4, unmodified Lys18, acetyl-Lys9, acetyl-Lys14, and acetyl-Lys23 peptides, but it reacts to two kinds of acetyl-Lys18 peptides (http://www.activemotif.com/catalog/details/39755/histone-h3-acetyl-lys18-antibody-pab-3). Anti-FLAG (M2) was purchased from Sigma. Normal rabbit and mouse IgG (Santa Cruz Biotechnology) were used as a control.

For flow cytometric analysis of murine samples, PE/Cy7 anti-CD3 (145-2C11), PerCP-Cy5.5 anti-CD4 (RM4-5), APC/Cy7 or FITC anti-CD8a (53–6.7), APC anti-TIGIT (1G9) and PE anti-CD226 (10E5) antibodies were purchased from BioLegend; PE anti-CD45R/B220 (RA3-6B2), FITC anti-CD11c (HL3), PE anti-PD-1 (J43), PE anti-IFN-γ (#554412) and PE anti-PD-1 (#551892) antibodies were purchased from BD Bioscience; and eFluor450 anti-Foxp3 (FJK-16s), PE anti-IL-10 (JES5-16E3), Biotin anti-CD90.1 (HIS51) and PE anti-Blimp1 antibodies were purchased from eBioscience. For flow cytometric analysis of human samples, PerCP-Cy5.5 anti-CD4 (OKT4) was purchased from eBioscience, and APC anti-TIGIT (741182) antibodies was purchased from R&D systems. Brilliant Violet421 anti-IL-10 and FITC-labeled goat anti-mouse IgG were purchased from BioLegend. Anti-HTLV I gp46 (67/5.5.13.1) was purchased from Abcam. Biotin conjugated anti-CADM1 (3E1) antibody was generated as described [55]. PE and PE/Cy7 streptavidin were purchased from BD Bioscience. The Near-IR LIVE/DEAD Fixable Dead Cell Stain Kit was purchased from Invitrogen. APC labeled mouse IgG and PE labeled rat IgG were purchased from eBioscience. Brilliant Violet 421 labeled rat IgG was purchased from BioLegend. Mouse IgG (MOPC-21) was purchased from Sigma for isotype controls. For functional analysis of patient samples, anti-TIGIT antibody (MBSA43) was purchased from eBioscience. Anti-PD-1 antibody (EH12.2H7) and mouse IgG1 isotype control (MOPC-21) were purchased from BioLegend.

### ELISA

CD4^+^ T cells from non-Tg and HBZ-Tg mice were enriched by CD4 magnetic particles (BD) and then seeded into a 96-well plate at 1 or 2x10^5^ cells and cultured for the indicated time. Supernatants from cultured cells were centrifuged to remove debris and IL-10 was then measured using QuantikineELISA from R&D systems according to the manufacturer’s protocol.

### ELISPOT assay

Mice were immunized with bacterially generated recombinant Tax protein and CpG by subcutaneous inoculation at days 0 and 14, and splenocytes were collected at day 21. Splenocytes of immunized mice and human PBMCs were subjected to ELISPOT assay as described [56]. Recombinant mouse TIGIT-Fc and mouse lgG2A-Fc (R&D) were coupled to Dynabeads M-450 (Invitrogen) according to the manufacturer’s protocol. Cells were stimulated with Tax peptides along with the Fc-conjugated beads or blocking antibodies as indicated.

### Cytometric bead array

Splenocytes from non-Tg and HBZ-Tg mice were stimulated with LPS overnight, followed by sorting of dendritic cells using FACS AriaII (BD). Sorted cells were seeded into a 96-well plate at 1 x 10^6^/ml and cultured in the presence of LPS for 24 hours. Supernatants were centrifuged to remove debris and IL-10 was then measured using IL-10 Enhanced Sensitivity Flex Set from BD according to the manufacturer’s protocol.

### HTLV-1 infection to primary CD4^+^ T cells

Human CD4^+^ cells were collected using Human CD4 T Lymphocyte Enrichment Set (BD) and then stimulated with PHA (3 μg/ml) in the presence of IL-2 (150 U/ml) for 3 days, followed by co-cultured with irradiated MT-2 for 3 days. Infected primary CD4^+^ T cells were detected by anti-HTLV-1 gp46 (Env) followed by FITC-labeled anti-mouse IgG.

### Knockdown of HBZ

ATL- 43T(-) cells were transfected with short interfering RNA (siRNA) twice at 0 and 48 hours using Lipofectamin2000 as described [57]. Over 90% cells were successfully transfected and they were harvested at 72 hours for RNA extraction.

### Statistical analysis

The Mann-Whitney test using GraphPad Prism software or the Student’s t test was used to determine significance where appropriate. All statistical analyses are shown as *P < 0.05 and **P < 0.01.

### Data access

All raw sequence data were deposited in the DNA Data Bank of Japan (DDBJ) under the accession number DRA003229 and DRA003744.

## Supporting Information

S1 Fig HTLV-1 newly infected cells expressed higher level of TIGIT. Human primary CD4^+^ T cells were stimulated with PHA (3μg/ml) for 3 days and then co-cultured with irradiated HTLV-1 infected cell line MT-2 for 3 days. Expression of TIGIT was analyzed by FCM in HTLV-1 infected Env^+^CD4^+^ T cells.
(PPTX)

S2 Fig Expression of TIGIT was suppressed by HBZ knockdown. HBZ was knock-down by siRNA in an ATL cell line, 43T(-). Expression levels of TIGIT were analyzed by realtime PCR. Results shown are the mean ± SD in triplicate. *P < 0.05.
(PPTX)

S3 Fig Transcriptional level of wt HBZ and mutant HBZ. Expression levels of HBZ-deletion mutants in luciferase assays (Fig 3B, 3C and 3D) were analyzed by realtime PCR using primers for the common sequence of all plasmids at SRα region (A for Fig 3B, B for Fig 3C and C for Fig 3D). RNA was extracted from simultaneously transfected and stimulated cells with luciferase assays. Results shown are the mean ± SD in triplicate.
(PPTX)

S4 Fig Blimp1 protein expressed higher level in CD4^+^ T cells of HBZ-Tg than those of non-Tg. Flow cytometry analysis of Blimp1 on CD4^+^ T cells from non-Tg and HBZ-Tg mouse. Splenocytes were stimulated with plate-coated anti-CD3 (1 μg/ml) and soluble anti-CD28 (1 μg/ml).
(PPTX)

S5 Fig IL-10 production of HTLV-1 infected cells in HAM/TSP patients. PBMCs from HAM/TSP patients (n = 5) and healthy donors (n = 3) were stimulated with PMA/ionomycin for 4 hours in the presence of brefeldin A. Expression of IL-10 were analyzed by FCM levels. IL-10 MFI of stained with anti-IL10 and isotype control were shown in the upper right.
(PPTX)

S6 Fig Transcriptional levels of *TIGIT*, *Fgl2*, *Blimp1 and Il10* genes in ATL cases. Expression levels of *TIGIT*, *Fgl2*, *Blimp1* and *IL-10* were analyzed by realtime PCR in PBMCs from ATL patients (n = 10–13) and in CD4^+^ T cells from HD (n = 4).
(PPTX)

S7 Fig Expression of C/EBPα in the presence of HBZ. Expression level of C/EBPα in luciferase assays (Fig 5C) were analyzed by realtime PCR. RNA was extracted from simultaneously transfected cells with luciferase assays. Results shown are the mean ± SD in triplicate. The representative result was shown for two independent experiments.
(PPTX)

S8 Fig CD226 expression in mice and human. Expression levels of *Cd226* were analyzed by realtime PCR in CD4^+^ T cells from non-Tg (n = 4), TIGIT^+^CD4^+^ and TIGIT^-^CD4^+^ T cells from HBZ-Tg (n = 3). Expression levels of *CD226* were analyzed by realtime PCR in CD4^+^ T cells from HD (n = 4) and ATL patients (n = 10).
(PPTX)

S9 Fig PD-1 expression on CD4^+^ T cells of HBZ-Tg mice. Expression levels of PD-1 were analyzed by FCM in CD4^+^ T cells from non-Tg (n = 4) and HBZ-Tg (n = 4) mice. Representative histograms were shown. *P < 0.05.
(PPTX)

S1 Table Genes upregulated by HBZ (Log_2_ fold > 2.9). (DOCX)

S2 Table Genes downregulated by HBZ (Log_2_ fold < -2.5). (DOCX)

S3 Table Reads and peaks of ChIP-seq analyses using HBZ transduced primary mouse T cells. (DOCX)

S4 Table Enriched gene promoters by HBZ-Flag-ChIP-seq. (DOCX)

S5 Table Percentages of TIGIT and/or PD-1 positivity in CD8^+^ T cells of HAM/TSP cases. The numbers indicate the percentage of CD8^+^ T cells. The mean percentages (± SD) from 4 donors are shown for healthy donor (HD).
(DOCX)

S6 Table Primers used in this study. (DOCX)
