# Supplementary material for: HTLV-1 bZIP Factor Impairs Anti-viral Immunity by Inducing Co-inhibitory Molecule, T Cell Immunoglobulin and ITIM Domain (TIGIT)
Source: PLoS Pathog. 2016 Jan 6;12(1):e1005372. doi: 10.1371/journal.ppat.1005372 (PMC4703212; doi:10.1371/journal.ppat.1005372)
Supplement: S1 Fig — Human primary CD4+ T cells were stimulated with PHA (3μg/ml) for 3 days and then co-cultured with irradiated HTLV-1 infected cell line MT-2 for 3 days. Expression of TIGIT was analyzed by FCM in HTLV-1 infected Env+CD4+ T cells. (PPTX) [file ppat.1005372.s001.pptx]

## Slide 1
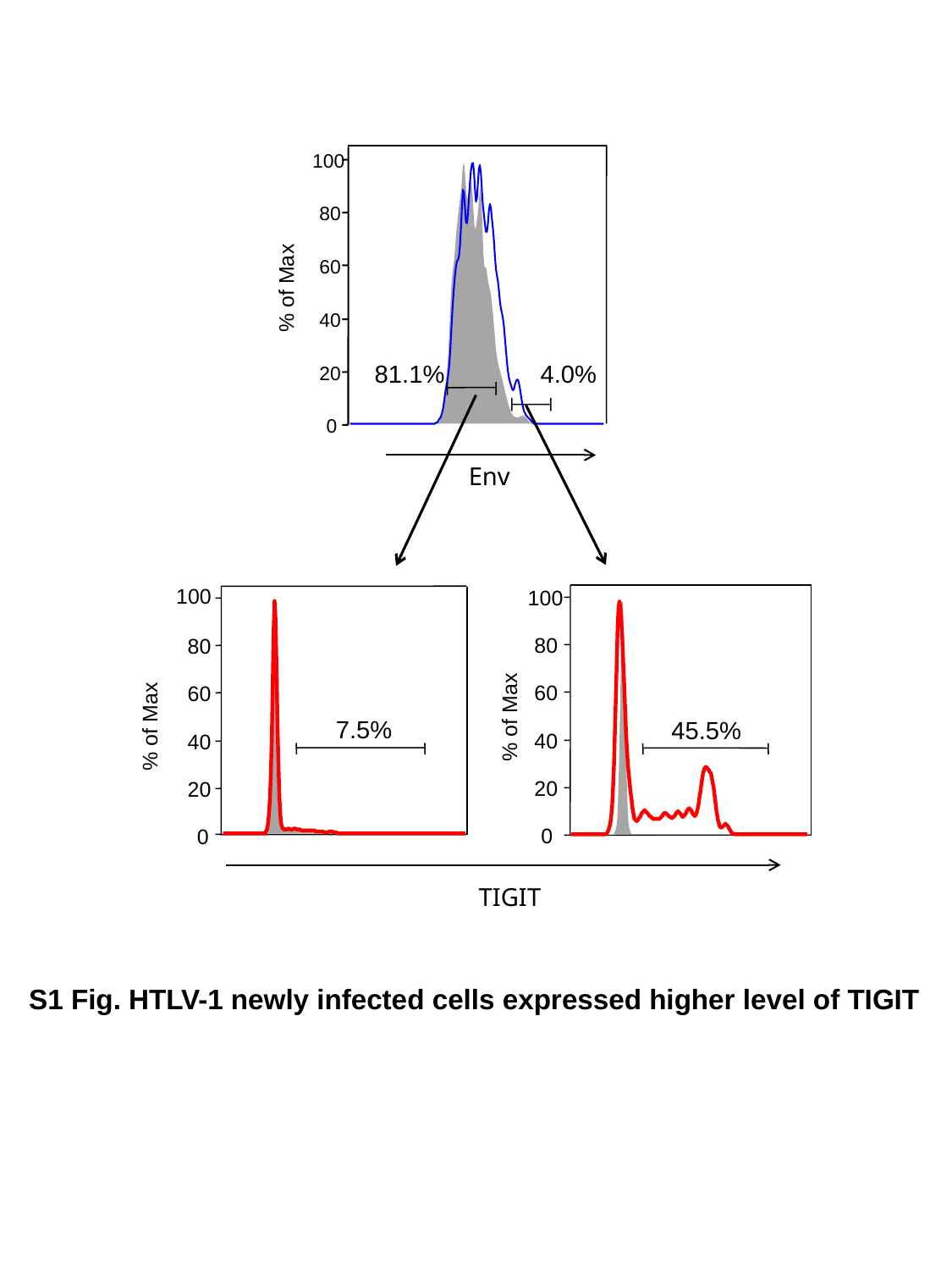

100
80
60
% of Max
40
4.0%
81.1%
20
0
Env
100
100
80
60
40
20
0
80
60
% of Max
% of Max
7.5%
45.5%
40
20
0
TIGIT
S1 Fig. HTLV-1 newly infected cells expressed higher level of TIGIT
