# Supplementary material for: HTLV-1 bZIP Factor Impairs Anti-viral Immunity by Inducing Co-inhibitory Molecule, T Cell Immunoglobulin and ITIM Domain (TIGIT)
Source: PLoS Pathog. 2016 Jan 6;12(1):e1005372. doi: 10.1371/journal.ppat.1005372 (PMC4703212; doi:10.1371/journal.ppat.1005372)
Supplement: S2 Fig — HBZ was knock-down by siRNA in an ATL cell line, 43T(-). Expression levels of TIGIT were analyzed by realtime PCR. Results shown are the mean ± SD in triplicate. *P < 0.05. (PPTX) [file ppat.1005372.s002.pptx]

## Slide 1
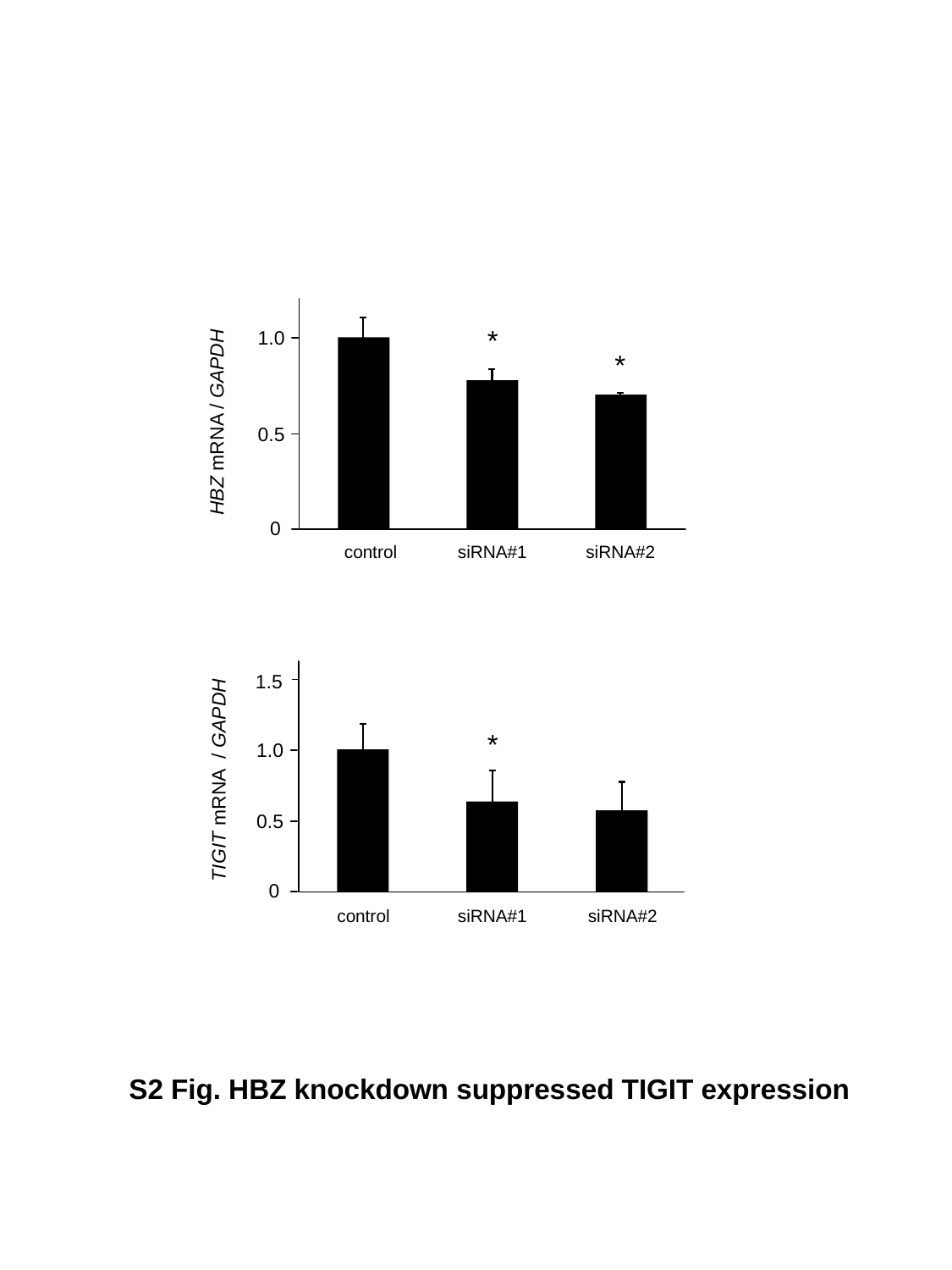

*
1.0
0.5
0
*
HBZ mRNA / GAPDH
control
siRNA#1
siRNA#2
1.5
1.0
0.5
0
control
siRNA#1
siRNA#2
*
TIGIT mRNA / GAPDH
S2 Fig. HBZ knockdown suppressed TIGIT expression
