# Supplementary material for: HTLV-1 bZIP Factor Impairs Anti-viral Immunity by Inducing Co-inhibitory Molecule, T Cell Immunoglobulin and ITIM Domain (TIGIT)
Source: PLoS Pathog. 2016 Jan 6;12(1):e1005372. doi: 10.1371/journal.ppat.1005372 (PMC4703212; doi:10.1371/journal.ppat.1005372)
Supplement: S3 Fig — Expression levels of HBZ-deletion mutants in luciferase assays (Fig 3B, 3C and 3D) were analyzed by realtime PCR using primers for the common sequence of all plasmids at SRα region (A for Fig 3B, B for Fig 3C and C for Fig 3D). RNA was extracted from simultaneously transfected and stimulated cells with luciferase assays. Results shown are the mean ± SD in triplicate. (PPTX) [file ppat.1005372.s003.pptx]

## Slide 1
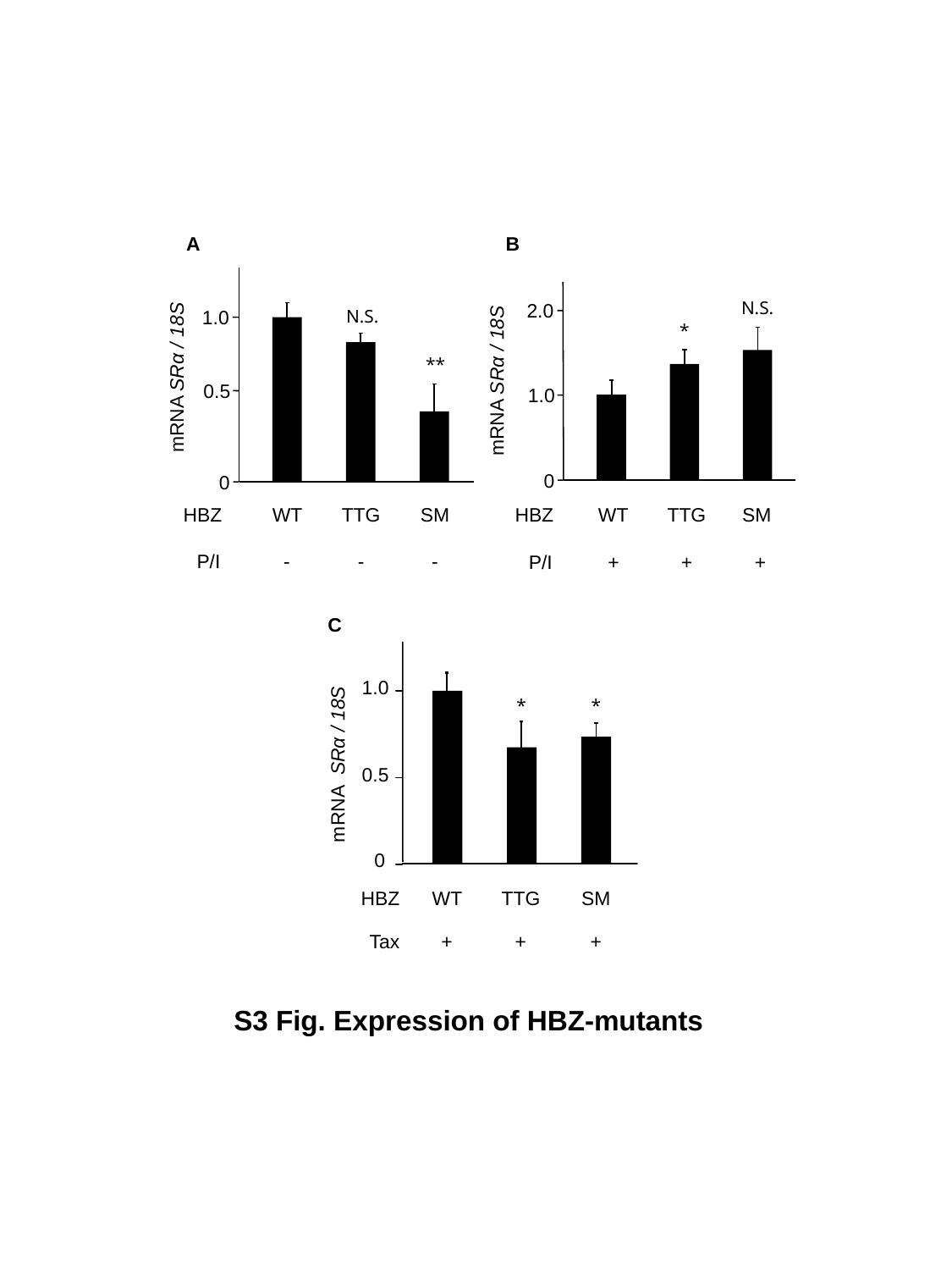

A
B
1.0
0.5
0
N.S.
2.0
1.0
N.S.
*
**
mRNA SRα / 18S
mRNA SRα / 18S
0
HBZ
WT
TTG
SM
HBZ
WT
TTG
SM
P/I
-
-
-
P/I
+
+
+
C
1.0
0.5
0
*
*
mRNA SRα / 18S
HBZ
WT
TTG
SM
Tax
+
+
+
S3 Fig. Expression of HBZ-mutants
