# Supplementary figures and images for: HTLV-1 bZIP Factor Impairs Anti-viral Immunity by Inducing Co-inhibitory Molecule, T Cell Immunoglobulin and ITIM Domain (TIGIT)
Source: PLoS Pathog. 2016 Jan 6;12(1):e1005372. doi: 10.1371/journal.ppat.1005372 (PMC4703212; doi:10.1371/journal.ppat.1005372)

## Slide 1
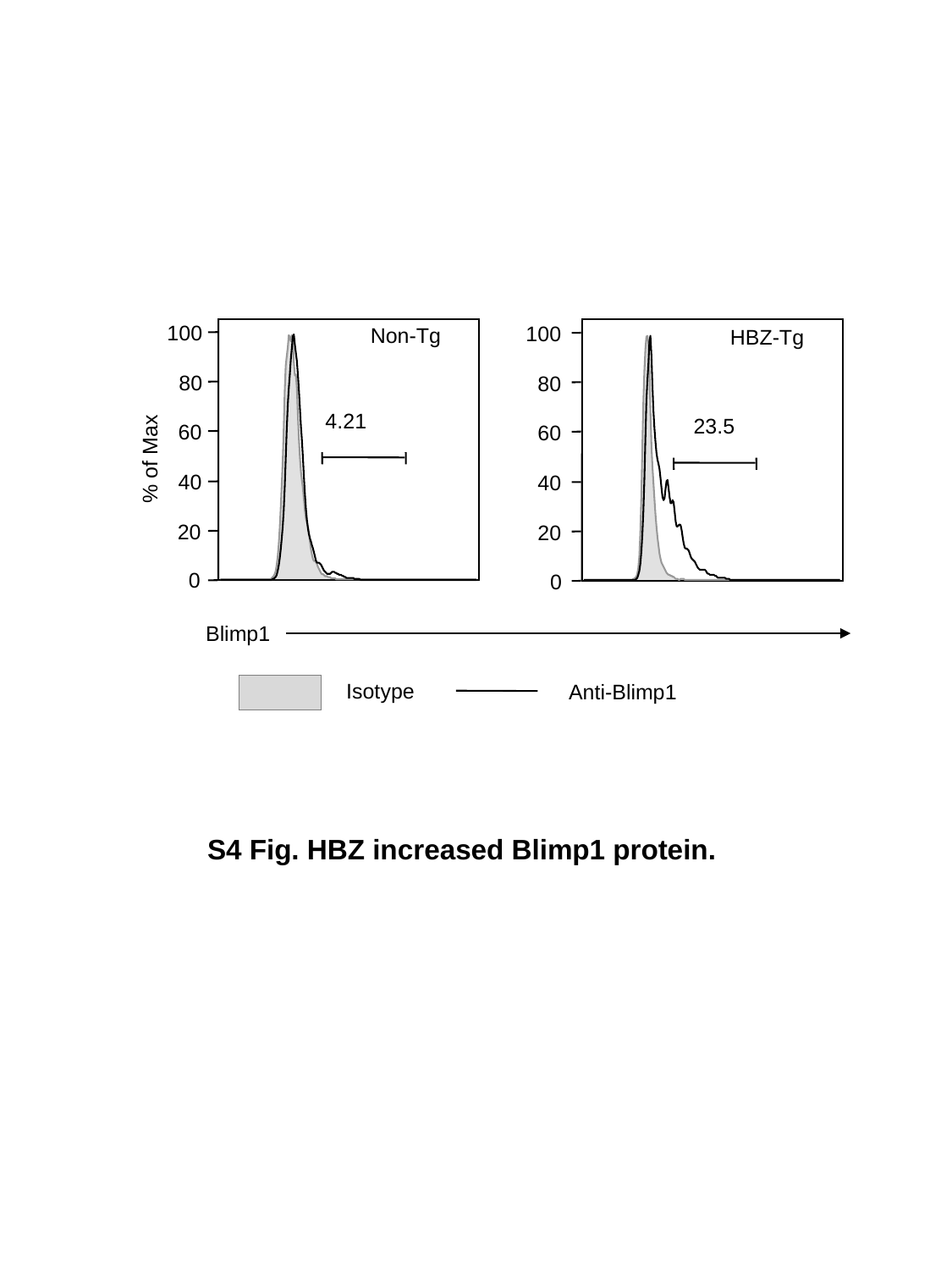

100
100
Non-Tg
HBZ-Tg
80
80
4.21
23.5
60
60
% of Max
40
40
20
20
0
0
Blimp1
Isotype
Anti-Blimp1
S4 Fig. HBZ increased Blimp1 protein.

Supplement: S4 Fig — Flow cytometry analysis of Blimp1 on CD4+ T cells from non-Tg and HBZ-Tg mouse. Splenocytes were stimulated with plate-coated anti-CD3 (1 μg/ml) and soluble anti-CD28 (1 μg/ml). (PPTX) [file ppat.1005372.s004.pptx]

## Slide 1
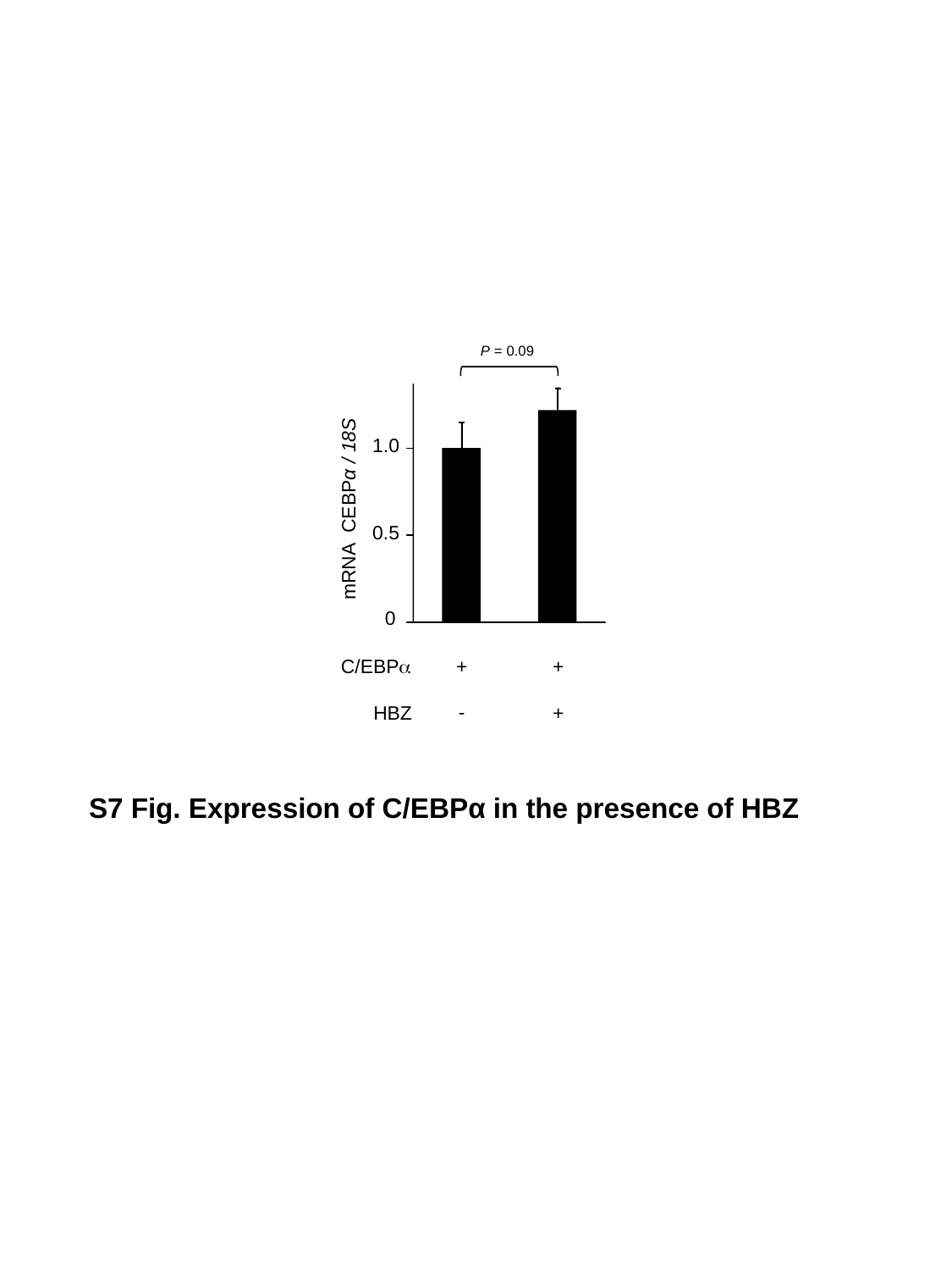

P = 0.09
1.0
0.5
0
mRNA CEBPα / 18S
C/EBPα
+
+
-
HBZ
+
S7 Fig. Expression of C/EBPα in the presence of HBZ

Supplement: S7 Fig — Expression level of C/EBPα in luciferase assays (Fig 5C) were analyzed by realtime PCR. RNA was extracted from simultaneously transfected cells with luciferase assays. Results shown are the mean ± SD in triplicate. The representative result was shown for two independent experiments. (PPTX) [file ppat.1005372.s007.pptx]
