# Supplementary material for: HTLV-1 bZIP Factor Impairs Anti-viral Immunity by Inducing Co-inhibitory Molecule, T Cell Immunoglobulin and ITIM Domain (TIGIT)
Source: PLoS Pathog. 2016 Jan 6;12(1):e1005372. doi: 10.1371/journal.ppat.1005372 (PMC4703212; doi:10.1371/journal.ppat.1005372)
Supplement: S5 Fig — PBMCs from HAM/TSP patients (n = 5) and healthy donors (n = 3) were stimulated with PMA/ionomycin for 4 hours in the presence of brefeldin A. Expression of IL-10 were analyzed by FCM levels. IL-10 MFI of stained with anti-IL10 and isotype control were shown in the upper right. (PPTX) [file ppat.1005372.s005.pptx]

## Slide 1
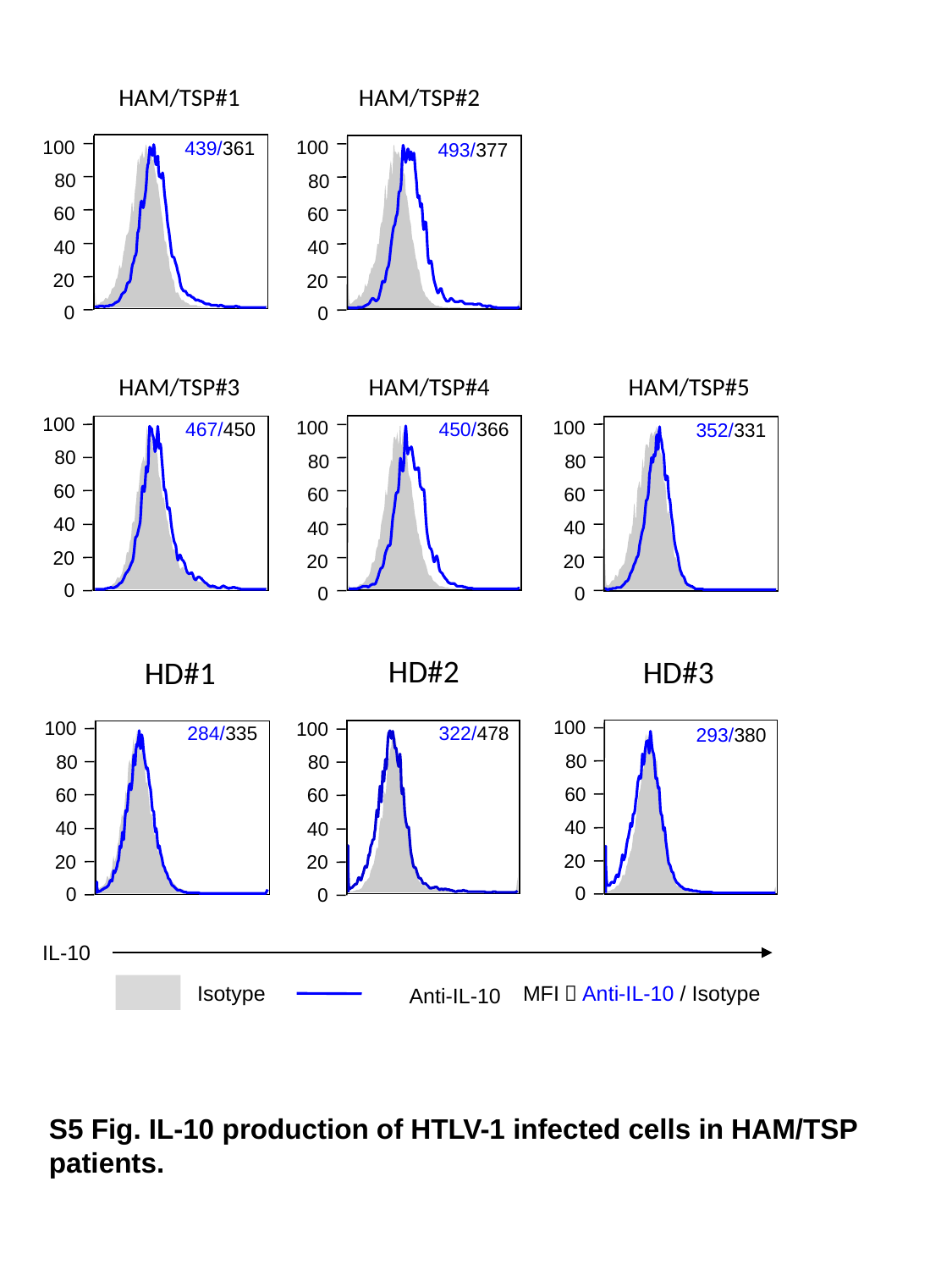

HAM/TSP#2
HAM/TSP#1
100
80
60
40
20
0
100
80
60
40
20
0
100
80
60
40
20
0
100
80
60
40
20
0
100
80
60
40
20
0
HD#2
HD#3
HD#1
100
80
60
40
20
0
100
80
60
40
20
0
100
80
60
40
20
0
IL-10
Isotype
Anti-IL-10
439/361
493/377
HAM/TSP#3
HAM/TSP#4
HAM/TSP#5
467/450
450/366
352/331
284/335
322/478
293/380
MFI：Anti-IL-10 / Isotype
S5 Fig. IL-10 production of HTLV-1 infected cells in HAM/TSP patients.
