# Supplementary material for: HTLV-1 bZIP Factor Impairs Anti-viral Immunity by Inducing Co-inhibitory Molecule, T Cell Immunoglobulin and ITIM Domain (TIGIT)
Source: PLoS Pathog. 2016 Jan 6;12(1):e1005372. doi: 10.1371/journal.ppat.1005372 (PMC4703212; doi:10.1371/journal.ppat.1005372)
Supplement: S6 Fig — Expression levels of TIGIT, Fgl2, Blimp1 and IL-10 were analyzed by realtime PCR in PBMCs from ATL patients (n = 10–13) and in CD4+ T cells from HD (n = 4). (PPTX) [file ppat.1005372.s006.pptx]

## Slide 1
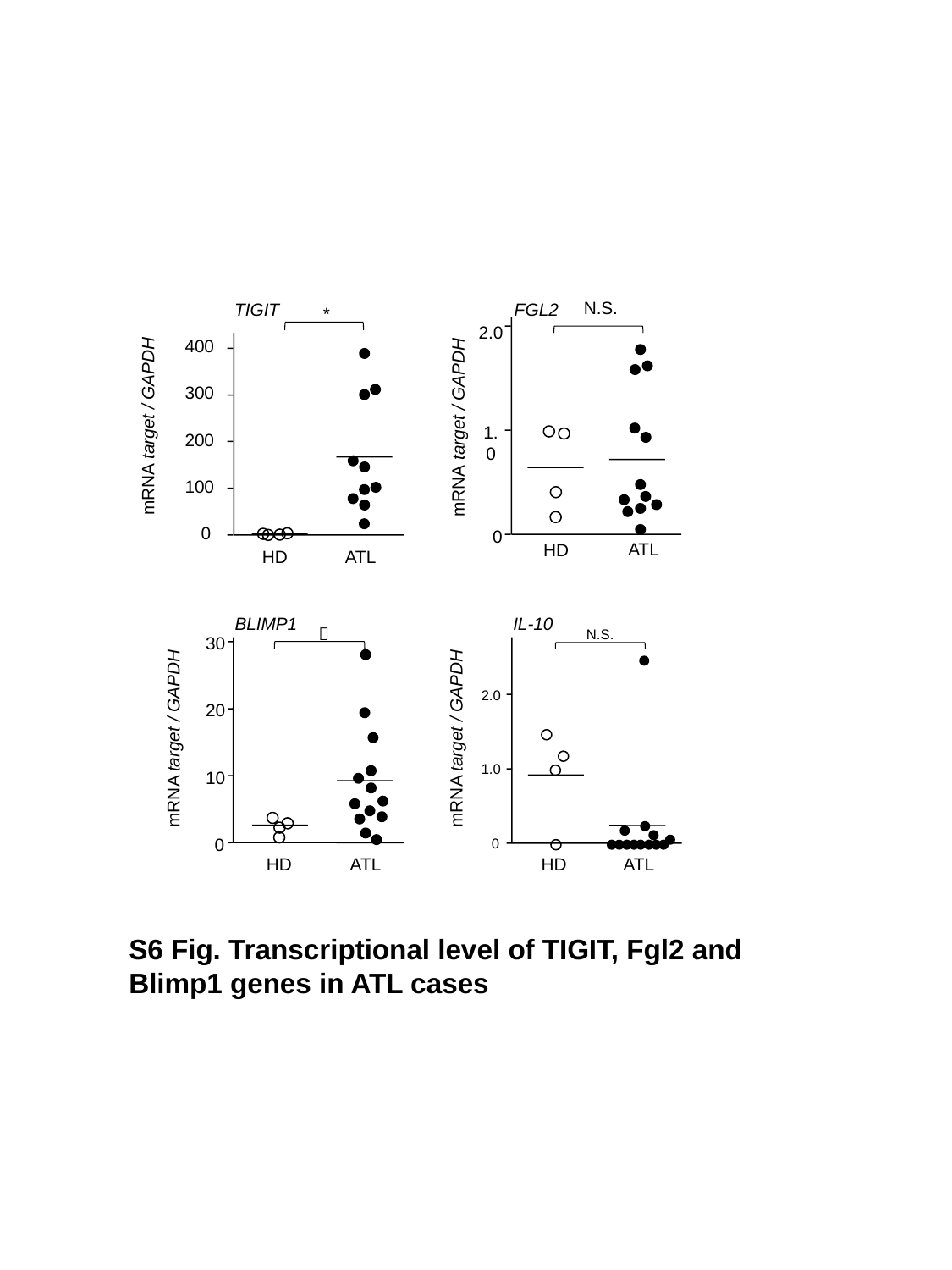

N.S.
*
TIGIT
FGL2
2.0
400
300
mRNA target / GAPDH
mRNA target / GAPDH
1.0
200
100
0
0
ATL
HD
ATL
HD
BLIMP1
IL-10
＊
N.S.
30
2.0
20
mRNA target / GAPDH
mRNA target / GAPDH
1.0
10
0
0
HD
ATL
HD
ATL
S6 Fig. Transcriptional level of TIGIT, Fgl2 and Blimp1 genes in ATL cases
