# Supplementary material for: HTLV-1 bZIP Factor Impairs Anti-viral Immunity by Inducing Co-inhibitory Molecule, T Cell Immunoglobulin and ITIM Domain (TIGIT)
Source: PLoS Pathog. 2016 Jan 6;12(1):e1005372. doi: 10.1371/journal.ppat.1005372 (PMC4703212; doi:10.1371/journal.ppat.1005372)
Supplement: S8 Fig — Expression levels of Cd226 were analyzed by realtime PCR in CD4+ T cells from non-Tg (n = 4), TIGIT+CD4+ and TIGIT-CD4+ T cells from HBZ-Tg (n = 3). Expression levels of CD226 were analyzed by realtime PCR in CD4+ T cells from HD (n = 4) and ATL patients (n = 10). (PPTX) [file ppat.1005372.s008.pptx]

## Slide 1
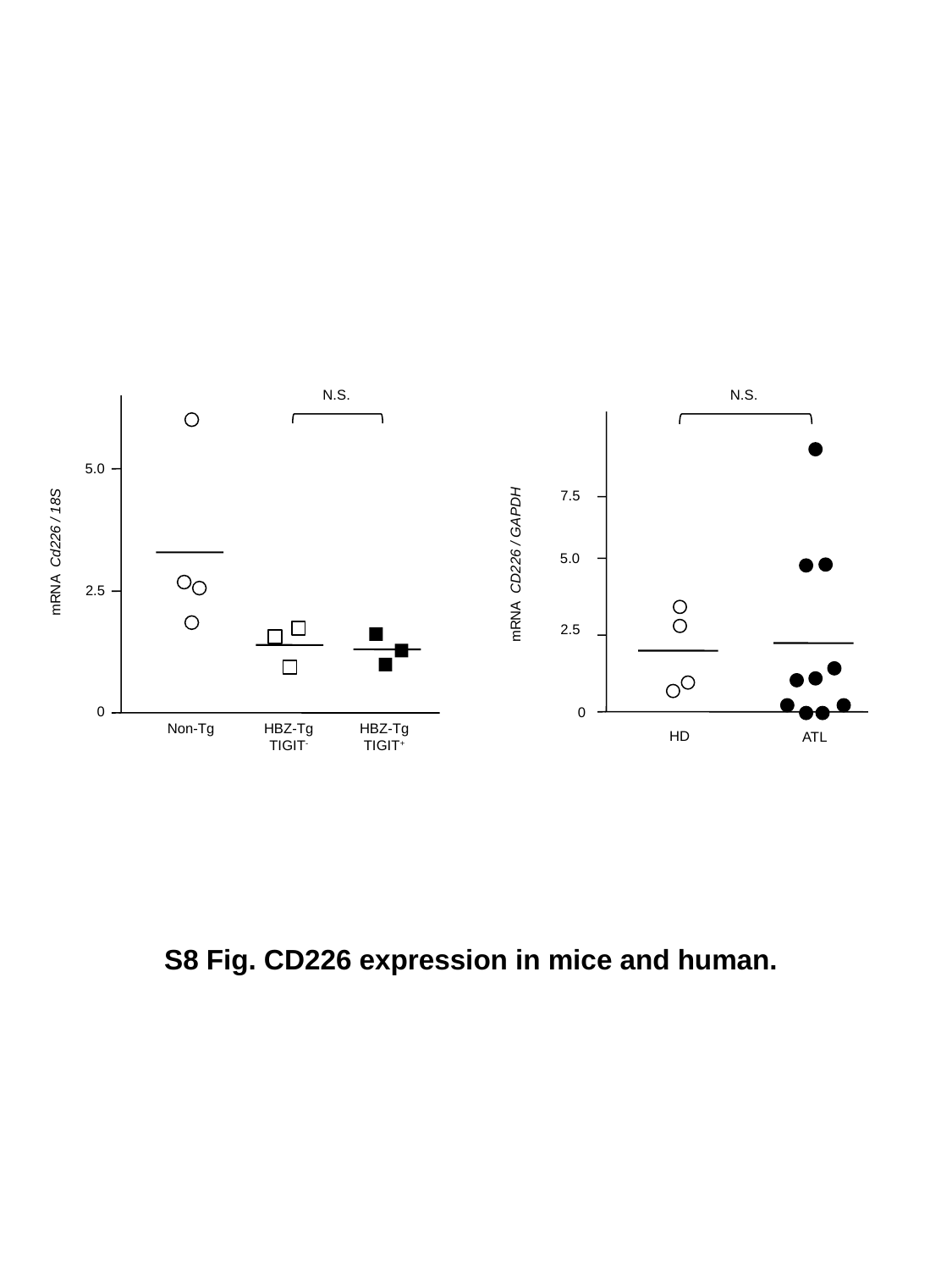

N.S.
7.5
5.0
2.5
0
HD
ATL
N.S.
5.0
mRNA Cd226 / 18S
2.5
0
Non-Tg
HBZ-Tg
TIGIT-
HBZ-Tg
TIGIT+
mRNA CD226 / GAPDH
S8 Fig. CD226 expression in mice and human.
