# Supplementary material for: HTLV-1 bZIP Factor Impairs Anti-viral Immunity by Inducing Co-inhibitory Molecule, T Cell Immunoglobulin and ITIM Domain (TIGIT)
Source: PLoS Pathog. 2016 Jan 6;12(1):e1005372. doi: 10.1371/journal.ppat.1005372 (PMC4703212; doi:10.1371/journal.ppat.1005372)
Supplement: S9 Fig — Expression levels of PD-1 were analyzed by FCM in CD4+ T cells from non-Tg (n = 4) and HBZ-Tg (n = 4) mice. Representative histograms were shown. *P < 0.05. (PPTX) [file ppat.1005372.s009.pptx]

## Slide 1
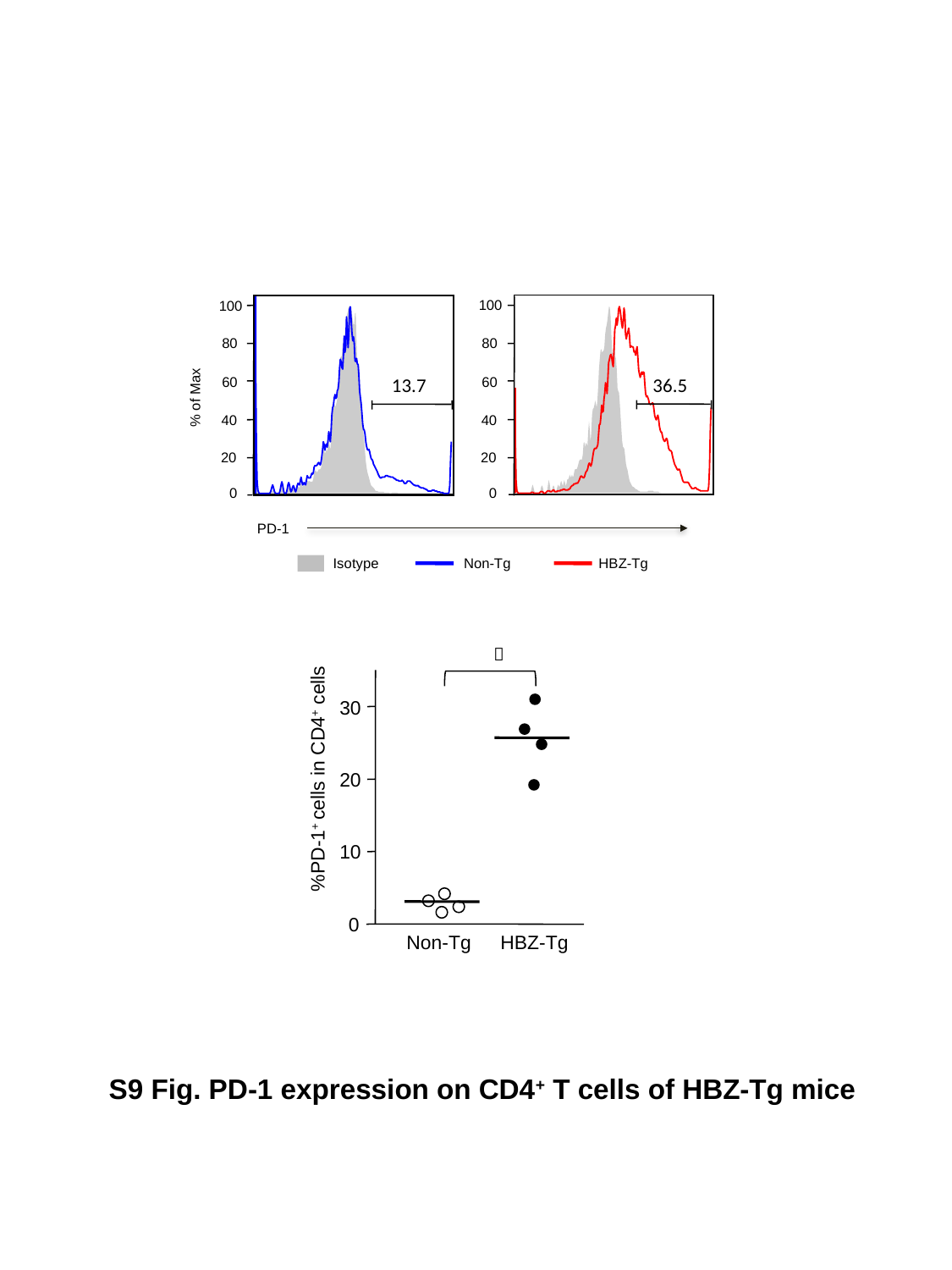

100
100
80
80
13.7
36.5
60
60
% of Max
40
40
20
20
0
0
PD-1
Isotype
Non-Tg
HBZ-Tg
＊
30
%PD-1+ cells in CD4+ cells
20
10
0
HBZ-Tg
Non-Tg
S9 Fig. PD-1 expression on CD4+ T cells of HBZ-Tg mice
