# Supplementary material for: HTLV-1 bZIP Factor Impairs Anti-viral Immunity by Inducing Co-inhibitory Molecule, T Cell Immunoglobulin and ITIM Domain (TIGIT)
Source: PLoS Pathog. 2016 Jan 6;12(1):e1005372. doi: 10.1371/journal.ppat.1005372 (PMC4703212; doi:10.1371/journal.ppat.1005372)
Supplement: S1 Table — (DOCX) [file ppat.1005372.s010.docx]

**S1 Table. Genes upregulated by HBZ (Log_2_ fold > 2.9).**

| **Gene Symbol** | **Refseq ID** | **Log2 Ratio** |
| --- | --- | --- |
| Tnfrsf13c | NM_028075 | 5.594 |
| Dnase1l3 | NM_007870 | 5.511 |
| Cd83 | NM_009856 | 5.079 |
| Angptl2 | NM_011923 | 4.699 |
| Sqrdl | NR_027888 | 4.698 |
| Armcx6 | NM_001007578 | 4.576 |
| Tigit | NM_001146325 | 4.542 |
| Cd24a | NM_009846 | 4.504 |
| H2-Aa | NM_010378 | 4.488 |
| Tspan2 | NM_001243132 | 4.269 |
| Fam213a | NM_027464 | 4.245 |
| Il4 | NM_021283 | 4.205 |
| Aqp11 | NM_175105 | 4.204 |
| Armcx2 | NM_001166398 | 3.899 |
| Nrp1 | NM_008737 | 3.812 |
| Tulp3 | NM_011657 | 3.798 |
| Marcksl1 | NM_010807 | 3.758 |
| Rpph1 | NR_002142 | 3.709 |
| Nckap1 | NM_016965 | 3.591 |
| Fam65c | NM_001080708 | 3.522 |
| Ppp2r3a | NM_001161362 | 3.392 |
| Foxp3 | NM_001199348 | 3.364 |
| Snora21 | NR_028078 | 3.41191 |
| Hdac7 | NM_001204280 | 3.25921 |
| Slc4a8 | NM_021530 | 3.24083 |
| Tfg | NM_001252443 | 3.21721 |
| Clock | NM_007715 | 3.20775 |
| Gstt3 | NM_133994 | 3.20443 |
| Hic1 | NM_001098203 | 3.19979 |
| Asb2 | NM_023049 | 3.19302 |
| Pdcd1 | NM_008798 | 3.18566 |
| Lzts1 | NM_199364 | 3.07988 |
| Il10 | NM_010548 | 3.05316 |
| Ccr5 | NM_009917 | 3.04177 |
| Prmt1 | NM_001252477 | 3.01252 |
| Rpl22 | NM_001277114 | 2.99252 |
| Shmt2 | NM_028230 | 2.988 |
| Nt5dc2 | NM_027289 | 2.97937 |
| Cobll1 | NM_027225 | 2.9768 |
| Pxmp2 | NM_008993 | 2.97538 |
| Tcf12 | NM_001253865 | 2.96159 |
| Larp1b | NM_001040399 | 2.93742 |
| Tnfrsf8 | NM_009401 | 2.93535 |
| Ccr4 | NM_009916 | 2.93294 |
