# Supplementary material for: HTLV-1 bZIP Factor Impairs Anti-viral Immunity by Inducing Co-inhibitory Molecule, T Cell Immunoglobulin and ITIM Domain (TIGIT)
Source: PLoS Pathog. 2016 Jan 6;12(1):e1005372. doi: 10.1371/journal.ppat.1005372 (PMC4703212; doi:10.1371/journal.ppat.1005372)
Supplement: S2 Table — (DOCX) [file ppat.1005372.s011.docx]

**S2 Table. Genes downregulated by HBZ (Log_2_ fold < -2.5).**

| **Gene Symbol** | **Refseq ID** | **Log2 Ratio** |
| --- | --- | --- |
| SHISA5 | NM_025858 | -3.819 |
| POU2AF1 | NM_011136 | -3.478 |
| HSD11B1 | NM_008288 | -3.327 |
| ETV3 | NM_001083318 | -3.280 |
| IFIT1 | NM_001101605 | -3.163 |
| Gzmb | NM_013542 | -3.139 |
| CYP11A1 | NM_019779 | -3.084 |
| ARL4D | NM_025404 | -3.074 |
| Apol7e (includes others) | NM_001024848 | -3.065 |
| Ly6a (includes others) | NM_001099217 | -3.013 |
| HMP19 | NM_008741 | -2.974 |
| DAPL1 | NM_029723 | -2.914 |
| HBS1L | NM_019702 | -2.815 |
| SYTL2 | NM_001040085 | -2.813 |
| WASH1 | NM_026833 | -2.799 |
| ABLIM1 | NM_001103177 | -2.798 |
| S100A6 | NM_011313 | -2.798 |
| RBPJ | NM_009035 | -2.756 |
| NFU1 | NM_001170591 | -2.708 |
| KCTD10 | NM_001159941 | -2.692 |
| H60b/H60c | NM_001177775 | -2.635 |
| SORL1 | NM_011436 | -2.598 |
| CD226 | NM_178687 | -2.579 |
| PGPEP1L | NM_030101 | -2.577 |
| WSCD2 | NM_177292 | -2.536 |
| Ms4a4b (includes others) | NM_029499 | -2.531 |
| PDE2A | NM_001243757 | -2.526 |
| PENK | NM_001002927 | -2.523 |
