# Supplementary material for: HTLV-1 bZIP Factor Impairs Anti-viral Immunity by Inducing Co-inhibitory Molecule, T Cell Immunoglobulin and ITIM Domain (TIGIT)
Source: PLoS Pathog. 2016 Jan 6;12(1):e1005372. doi: 10.1371/journal.ppat.1005372 (PMC4703212; doi:10.1371/journal.ppat.1005372)
Supplement: S3 Table — (DOCX) [file ppat.1005372.s012.docx]

**S3 Table. Reads and peaks of ChIP-seq analyses using HBZ transduced primary mouse T cells.**

|  | Mock transduction | | | HBZ transduction | | |  |  |
| --- | --- | --- | --- | --- | --- | --- | --- | --- |
|  | Anti-AcH3 | Input | Isotype | Anti-AcH3 | Input | Isotype |  |  |
| # of total reads | 33,560,172 | 76,059,332 | 54,961,308 | 53,395,684 | 52,710,558 | 53,541,762 |  |  |
| # of mapped reads | 29,224,420 | 59,179,510 | 42,179,882 | 42,549,534 | 41,012,850 | 41,095,933 |  |  |
| # of peaks | 26,522 |  |  | 27,267 |  |  |  |  |
|  |  |  |  |  |  |  |  |  |
|  | Mock transduction | | | | HBZ transduction | | | |
|  | Anti-AcH3K9 | Anti-AcH3K18 | Anti-AcH3K27 | Input | Anti-AcH3K9 | Anti-AcH3K18 | Anti-AcH3K27 | Input |
| # of total reads | 10,907,495 | 20,582,731 | 26,673,484 | 50,935,380 | 10,395,573 | 21,194,969 | 35,275,392 | 49,078,385 |
| # of mapped reads | 9,700,769 | 18,242,551 | 23,564,507 | 39,817,927 | 9,220,037 | 18,879,056 | 31,273,563 | 38,752,005 |
| # of peaks | 24,189 | 38,256 | 29,254 |  | 21,806 | 36,749 | 33,109 |  |
|  |  |  |  |  |  |  |  |  |
|  |  |  |  |  |  |  |  |  |
|  | HBZ-FLAG transduction | |  |  |  |  |  |  |
|  | Anti-FLAG | Input |  |  |  |  |  |  |
| # of total reads | 31,824,673 | 30,184,437 |  |  |  |  |  |  |
| # of mapped reads | 26,455,353 | 26,204,482 |  |  |  |  |  |  |

Statistics of the tags generated for experiment are summarized. The numbers of reads and peaks are shown.
