# Supplementary material for: HTLV-1 bZIP Factor Impairs Anti-viral Immunity by Inducing Co-inhibitory Molecule, T Cell Immunoglobulin and ITIM Domain (TIGIT)
Source: PLoS Pathog. 2016 Jan 6;12(1):e1005372. doi: 10.1371/journal.ppat.1005372 (PMC4703212; doi:10.1371/journal.ppat.1005372)
Supplement: S4 Table — (DOCX) [file ppat.1005372.s013.docx]

**S4 Table. Enriched gene promoters by HBZ-Flag-ChIP-seq.**

| **ID** | **Gene Symbol** | **-10*LOG10 (pvalue)** |
| --- | --- | --- |
| NM_153553 | Npas4 | 186.7 |
| NM_007583 | Cacng2 | 145.04 |
| NR_029827 | Mir7b | 113.66 |
| NM_026244 | Slc39a9 | 77.92 |
| NM_007951 | Erh | 77.92 |
| NM_011579 | Tgtp1 | 69.96 |
| NM_030207 | Sfi1 | 68.32 |
| NM_028065 | Cnpy3 | 65.03 |
| NM_019446 | Barhl1 | 64.52 |
| NR_029794 | Mir212 | 62.92 |
| NR_029546 | Mir132 | 62.92 |
| NM_010696 | Lcp2 | 59.98 |
| NM_001146325 | Tigit | 59.87 |
| NM_025421 | Acyp1 | 59.08 |
| NM_172252 | Mrpl21 | 58.3 |
| NM_009212 | Ighmbp2 | 58.3 |
| NM_130893 | Scrt1 | 57.28 |
| NM_008972 | Ptma | 57.1 |
| NR_045997 | 1700113A16Rik | 56.61 |
| NM_153505 | Nckap1l | 56.27 |
| NM_001110305 | Keap1 | 56.27 |
| NM_001243138 | Gm13247 | 55.77 |
| NM_001252347 | Rundc3a | 55.47 |
| NM_008910 | Ppm1a | 54.84 |
| NM_001081954 | Dux | 54.16 |
| NM_001256087 | Rnf6 | 52.84 |
| NM_001033713 | Mef2a | 52.84 |
| NM_001253862 | Tcf12 | 52.56 |
| NM_001122667 | Mkl2 | 52.4 |
| NM_020507 | Tob2 | 51.69 |
| NM_007517 | Aup1 | 51.62 |
| NM_028036 | Tmco6 | 51.16 |
| NR_002847 | Malat1 | 50.57 |
| NM_001198859 | Ctbp1 | 50.56 |
| NM_011341 | Sdf4 | 50.31 |
| NM_080445 | B3galt6 | 50.31 |
| NM_001146707 | Nap1l1 | 50.14 |
