# Supplementary material for: HTLV-1 bZIP Factor Impairs Anti-viral Immunity by Inducing Co-inhibitory Molecule, T Cell Immunoglobulin and ITIM Domain (TIGIT)
Source: PLoS Pathog. 2016 Jan 6;12(1):e1005372. doi: 10.1371/journal.ppat.1005372 (PMC4703212; doi:10.1371/journal.ppat.1005372)
Supplement: S5 Table — The numbers indicate the percentage of CD8+ T cells. The mean percentages (± SD) from 4 donors are shown for healthy donor (HD). (DOCX) [file ppat.1005372.s014.docx]

**S5 Table. Percentages of TIGIT and/or PD-1 positivity in CD8^+^ T cells of HAM/TSP cases.**

| **Disease** | **TIGIT^-^PD1^-^** | **TIGIT^+^PD1^-^** | **TIGIT^-^PD1^+^** | **TIGIT^+^PD1^+^** | **PVL（％）** |
| --- | --- | --- | --- | --- | --- |
| HAM/TSP #1 | 52.4 | 12.6 | 18.8 | 16.2 | 19.7 |
| HAM/TSP #2 | 48.9 | 32.1 | 4.39 | 14.6 | 13.0 |
| HAM/TSP #3 | 26.4 | 15.1 | 26.4 | 32.1 | 12.5 |
| HAM/TSP #4 | 72.2 | 12.4 | 5.43 | 10.0 | 4.0 |
| HAM/TSP #5 | 46.6 | 21.4 | 10.4 | 21.6 | 3.4 |
| HD | 73.1 ± 11.2 | 3.70 ± 2.04 | 10.1 ± 2.60 | 13.1 ± 8.79 |  |

*The numbers indicate the percentage of CD8^+^ T cells. The mean percentages (± SD) from 4 donors are shown for healthy donor (HD).
