# Supplementary material for: HTLV-1 bZIP Factor Impairs Anti-viral Immunity by Inducing Co-inhibitory Molecule, T Cell Immunoglobulin and ITIM Domain (TIGIT)
Source: PLoS Pathog. 2016 Jan 6;12(1):e1005372. doi: 10.1371/journal.ppat.1005372 (PMC4703212; doi:10.1371/journal.ppat.1005372)
Supplement: S6 Table — (DOCX) [file ppat.1005372.s015.docx]

**S6 Table. Primers used in this study.**

For realtime PCR（human）

| TIGIT Forward | GTTCCAGATTCCATTGCTT |
| --- | --- |
| TIGIT Reverse | CTCCTGTCCAGCTGATTTT |
| CD226 Forward | GATGTTGGCTACTATTCCTGCTC |
| CD226 Reverse | CTGAACCACCTGTATCACCTTC |
| GAPDH Forward | GAGTCAACGGATTTGGTCGT |
| GAPDH Reverse | CGCTCCTGGAAGATGGTG |
| FGL2 Forward | CAGCCAAGAACAAATACAGTCACG |
| FGL2 Reverse | TGGGATCAGGTGTAACTCTGTAGG |
| BLIMP1 Forward | TCAAACTCAGCCTCTGTCCA |
| BLIMP1 Reverse | TCCAGCACTGTGAGGTTTCA |
| IL-10 Forward | GCCTAACATGCTTCGAGATC |
| IL-10 Reverse | TGATGTCTGGGTCTTGGTTC |
| CEBPα Forward | GACTTCTACGAGGCGGAGC |
| CEBPα Reverse | TGTAGGCGCTGATGTCGATG |
| SRα Forward | TCTGCGCCGTTACAGATCCAAG |
| SRα Reverse | AGTAACACTTCCGTACAGGCC |
| 18S Forward | GTAACCCGTTGAACCCCATT |
| 18S Reverse | CCATCCAATCGGTAGTAGCG |
| HBZ Forward | GTGAGCGCAAGTGGAGACAA |
| HBZ Reverse | TTATTGCAACCACATCGC |
| siHBZ#1 Forward | GCCAAACGAUAGGAAUCUUCU |
| siHBZ#1 Reverse | UCUUCUAAGGAUAGCAAACCG |
| siHBZ#2 Forward | CUGAGUAUUUGAAAAGGAAGG |
| siHBZ#2 Reverse | UUCCUUUUCAAAUACUCAGCG |
| si-control Forward | UUCUCCGAACGUGUCACGUUC |
| si-control Reverse | ACGUGACACGUUCGGAGAAAG |

For real-time PCR (mouse)

| Tigit Forward | **tcctggtgggatttacaagg** |
| --- | --- |
| Tigit Reverse | **aagcaaatgagtcccagcac** |
| Blimp1 Forward | GACGGGGGTACTTCTGTTCA |
| Blimp1 Reverse | GGCATTCTTGGGAACTGTGT |
| Il-10 Forward | ATTTGAATTCCCTGGGTGAGAAG |
| Il-10 Reverse | CACAGGGGAGAAATCGATGACA |
| Fgl2 Forward | GGTGCTCAAAGAAGTGCGGA |
| Fgl2 Reverse | GTTCCTGGACTCTACTGTCCTC |
| Cd226 Forward | AAGGAGCAACTGTAGCCAGG |
| Cd226 Reverse | acagtgaaactaaccctccaac |
| IL-12 Forward | TGGTTTGCCATCGTTTTGCTG |
| IL-12 Reverse | ACAGGTGAGGTTCACTGTTTCT |
| 18S Forward | GTAACCCGTTGAACCCCATT |
| 18S Reverse | CCATCCAATCGGTAGTAGCG |

For Luciferase-promoter (human)

| TIGIT-Luc Forward | tctcgagtgactgagttcaccaag |
| --- | --- |
| TIGIT-Luc Reverse | tGATATCagagggcctacaggaag |
| FGL2-Luc Forward | tttctcgagaaaggaggtggtttctctaactg |
| FGL2-Luc Reverse | tttagatcttgcgcagggctggag |

For ChIP-qPCR (mouse)

| TIGIT-promoter-F | AAGAGACGAGGGTCGGCAAAG |
| --- | --- |
| TIGIT-promoter-R | CTTTGGATGTCCCCTGGTGAGA |
| TIGIT-promoter-F1 | CCATCCCACCAGTGCACAA |
| TIGIT-promoter-R1 | AACCCAGGATGGGGAGGAA |
| TIGIT-promoter-F2 | ctggtgaaaagagggtctcg |
| TIGIT-promoter-R2 | tgtttccttcatggcctttc |
